# Sensitivity of the Dorsal-Central Retinal Pigment Epithelium to Sodium Iodate-Induced Damage Is Associated With Overlying M-Cone Photoreceptors in Mice

**DOI:** 10.1167/iovs.63.9.29

**Published:** 2022-08-26

**Authors:** Lili Lian, Yifan Zhai, Xuejiao Wan, Linxin Chen, Zuimeng Liu, Ruona Liu, Shijia Li, Jiajia Zhou, Yu Chen, Ling Hou, Huirong Li

**Affiliations:** 1 Laboratory of Developmental Cell Biology and Disease, School of Ophthalmology and Optometry and Eye Hospital, Wenzhou Medical University, Wenzhou, China; 2 State Key Laboratory of Ophthalmology, Optometry and Vision Science, Wenzhou Medical University, Wenzhou, China

**Keywords:** retinal pigment epithelium, geographic atrophy, retinal degeneration, dorsal-ventral axis, sodium iodate injury

## Abstract

**Purpose:**

Retinal pigment epithelium (RPE) degeneration is a leading cause of blindness in retinal degenerative diseases, but the mechanism of RPE regional degeneration remains largely unknown. This study aims to investigate the sensitivity of RPE to sodium iodate (SI) injury in the dorsal and ventral visual fields in mice and analyze whether overlaying cone photoreceptors regulate the sensitivity of RPE to SI-induced damage.

**Methods:**

SI was used to induce RPE degeneration in mice. Hematoxylin-eosin staining, immunostaining, and TUNEL assay were used to evaluate retinal degeneration along the dorsal-ventral axis. Flat-mounted and sectional retinal immunostaining were used to analyze the distribution of cones along the dorsoventral axis in C57BL/6, albino, and 129 mice. Electroretinography was used to examine the retinal function.

**Results:**

Dorsal-central RPE was more sensitive to SI-mediated injury along the dorsal-ventral axis in C57BL/6 mice. Compared with the ventral RPE, the dorsal-central RPE was dominantly covered by M cone photoreceptors in these mice. Interestingly, M cone photoreceptor degeneration was followed by dorsal RPE degeneration under a low dose of SI. Furthermore, the sensitivity of dorsal RPE to a low dose of SI was reduced in both albino and 129 mouse strains with dominant mixed cones instead of M cones in the dorsal visual field.

**Conclusions:**

These findings suggest that dorsal-central RPE is more sensitive to SI injury and that SI-induced RPE degeneration could be controlled by modifying the dominant overlying cone population in the mouse dorsal retina, thereby highlighting a potential role of M cones in RPE regional degeneration.

The retinal pigment epithelium (RPE) is a monolayer of polarized cobblestone-shaped post-mitotic cells that play a crucial role in the function and maintenance of the overlying photoreceptors (rod and cone).^1^^,^^2^ The rods and cones are highly specialized neurons that serve as the site of signal transduction where irradiant light is converted into neuronal signals in the eye.^3^ The interneurons (horizontal cells, bipolar cells, and amacrine cells) process and transduce the information to retinal ganglion cells (RGCs), which finally send the signals to the brain via the optic nerve for visual formation.^4^ During the visual processing, RPE has multiple functions to contribute to photoreceptor function, such as absorption of excess light, regeneration of vitamin A–derived chromophores to maintain the visual cycle, and release of trophic factors.^5^ Therefore physical disruption or functional impairment of RPE is thought to result in photoreceptor degeneration and eventually vision loss.^6^

Geographic atrophy (GA) is a typical pathological feature of dry age-related macular degeneration, showing degeneration of RPE and of secondary photoreceptor in the fovea, which results in loss of central vision. So far, no cure has been found for this condition.^6^^,^^7^ In the fovea, cones fill the foveal outer nuclear layer (ONL), whereas rods are absent from this area.^8^ Also, RGC density is higher in the fovea than in the rest of the retina.^9^ A consequence of this physical layout is that the fovea drives high spatial acuity vision and is responsible for bright-light and color vision, while the retinal periphery has low spatial acuity and is responsible for night-vision. As a supporting structure for photoreceptors, RPE also exhibits different features following their topography, such as high melanosome content in aging foveal RPE.^10^^–^^12^ Regarding gene expression profile, foveal RPE shows higher expression of chemokine-related genes, whereas peripheral RPE shows higher expression of proliferation-related genes.^13^ In addition, foveal RPE is more sensitive to damage by external stressors than peripheral RPE in age-related macular degeneration, which is the main cause of blindness among the elderly people.^7^ However, although RPE degeneration has been reported to be associated with multiple factors, including oxidative stress, inflammation, autophagy, lipid accumulation and loss of clearance ability, the mechanism underlying the differential vulnerability of the fovea to RPE degeneration is still unclear.^14^^–^^17^

Mice are a widely used animal model for studying human physiology and diseases. Concerning eye structure, mice have no typical fovea in the retina, but do possess an acute zone in the dorsal-temporal area of the retina where RGC density is higher.^18^ For mice, the perception of the visual field differs above and below the skyline.^19^ The lower visual field, imaged by the dorsal retina which shares some features with the human macula,^20^ often visualizes the ground. The upper visual field, imaged by the ventral retina, frequently perceives the sky to avoid predators.^21^ This retinal asymmetry is accompanied by a pronounced dorsal-ventral gradient in opsin (short [S] and middle [M] wavelength-specific opsin) expression across their cone photoreceptors.^22^ Indeed, whereas M opsin is dominant in the dorsal retina, which is involved in perceiving the ground, S opsin is dominant in the ventral retina, involved in detecting short-wavelength light from the sky.^23^ Moreover, the encoding of RGCs also differs between these two regions.^20^ Consistent with the asymmetric distribution of cones, the RPE should also shows varying features along the retina dorsal-ventral axis. Recently, it has been reported that the cell size and orientation show some differences between central and peripheral RPE.^24^ Additionally, differential expression of cell cycle ^25^ and adhesion related genes^26^ was also observed between central and peripheral RPE cells. Furthermore, the central RPE is more prone to be damaged by administration of sodium iodate (SI),^27^ a reagent used to induce a GA-like phenotype in rodents.^28^ These previous studies suggest that RPE cells have different features exist between RPE cells of the central and peripheral retina. However, rodent vision is functionally divided into dorsal and ventral fields. Therefore there is an urgent need to learn about the distinctive characteristics of the RPE at the dorsal and ventral retina, to better understand the pathological mechanisms mediating the differences in RPE degeneration between these two regions.

In the present study, we analyzed the unique features between the RPE of the dorsal and ventral retina of C57BL/6 mice upon different doses of SI. Our results show that dorsal-central RPE was more sensitive to SI-induced injury than the rest of the RPE in C57BL6 mice and suggest that this differential sensitivity is mediated by the overlying M cones at the dorsal retina.

## Materials and Methods

### Experimental Animals

Two-month-old adult C57BL/6 mice, 129 mice, and BALB/c (*Tyr^c^/Tyr^c^*) albino mice were purchased from Vital River Laboratory in Beijing, China. The mice were raised in the Wenzhou Medical University animal facility under standard 12-hour light/12-hour dark conditions (light period: 7 AM to 7 PM) and provided standard diet and tap water. All experiments were performed in accordance with the ARVO (Association for Research in Vision and Ophthalmology) statement for the Use of Animals and the approved guidelines of the Wenzhou Medical University Institutional Animal Care and Use Committee (permit number: WZMCOPT- 090316).

### Systemic SI Injection

SI (NalO_3_, S4007; Sigma-Aldrich Corp., St. Louis, MO, USA) was freshly dissolved in sterile saline and injected through the tail vein to generate a GA-like experimental model in mice. For tail vein injection, high (25 mg/kg) and low (15 mg/kg) doses of SI were used to generate severe retinal degeneration and selectively dorsal retinal degeneration, respectively. For intravitreal injection, 0.5 µL of SI (5 mg/mL) was injected into the eye of two-month-old C57BL/6 mice.

### Hematoxylin-Eosin Staining

Hematoxylin-eosin (H&E) staining has been described previously.^29^ Briefly, a mark was made at a 12 o'clock position of the cornea, and the extracted eyeballs were incubated in a mixed fixing solution (formaldehyde, ethanol, H_2_O glacial acetic acid; 1:4:4:1) for at least one day at room temperature. Paraffin retinal sections (5 µm) were stained by a standard program. The thickness of the outer nuclear layer was measured at a distance between 100 µm and 1000 µm from the optic nerve head to the peripheral retina.

### Quantitative RT-PCR

RPE patches were isolated as previously described.^30^ Briefly, retinas were dissected in DMEM and divided in dorsal and ventral parts, which were separately digested by hyaluronidase (Sigma-Aldrich; 1 mg/mL) incubation at 37°C for 40 minutes and then transfer to Hanks solution on ice for 30 minutes. The neural retinas were isolated and removed while immersed in the Hanks solution. The RPE patches were harvested by shaking the dorsal or ventral RPE and spun in a centrifuge at 2000 rpm for 10 minutes. RPE mRNA was extracted by Trizol reagent (Invitrogen, Carlsbad, CA, USA) according to the manufacturer's protocol. Quantitative real time PCR was performed on a 7500 Real-Time PCR Detection System (Applied Biosystems) with Power SYBR Green PCR Master Mix as previously described.^29^ All primer sequences used for real-time PCR are listed in the supplementary data (Supplementary Table S1).

### Western Blot Analysis

RPE sheets isolated from two-month-old C57BL/6 mice were lysed in a solution containing RIPA buffer (Beyotime Institute of Biotechnology, Jiangsu, China) and protease inhibitors (Beyotime Institute of Biotechnology). After sonication, protein samples were separated by 10% SDS-PAGE and transferred to PVDF membranes (Bio-Rad Life Science, Hercules, CA, USA) as described previously.^31^ The membranes were blocked for two hours in a blocking buffer containing 5% non-fat milk in PBS at room temperature and incubated overnight with primary antibodies at 4°C. Primary antibodies were anti-MITF (1:1000, ab12039; Abcam, Cambridge, MA, USA) and anti-GAPDH (KC-5GC, 1:5000; Aksomics, Shanghai, China). After washing with Tris-buffered saline solution containing Tween 20, the membranes were incubated with fluorescein-conjugated secondary antibodies (LI-COR Biosciences, Lincoln, NE, USA) at room temperature for two hours and analyzed by the Odyssey CLx System (LI-COR Biosciences).

### Electroretinography

Electroretinography was performed as previously described.^32^ Mice were dark-adapted overnight (≥12 hours) and anesthetized under dim red light. After five minutes of dilation, mice were stimulated by green (511 nm) or ultraviolet (UV, 363 nm) light-emitting diodes for detecting M- and S-cone–mediated responses, respectively. The optimal strength of green light was set at 0.75 candlepower sec/m^2^ and that of UV at 3 mW-s/m^2^. For the rod response, mice were stimulated by flash light at the intensity of 0.01 scotopic candlepower sec/m^2^ in a Ganzfeld dome (Roland Q400, Wiesbaden, Germany).

### Immunostaining and TUNEL Analysis

Eyes were dissected in DMEM, and a mark was made at the 12 o'clock position. For sectional immunostaining, the anterior segment of the eyeball was removed, and the rest of the eyeball was fixed in 4% paraformaldehyde (Sigma-Aldrich) at 4°C for two hours and then dehydrated in 30% sucrose at 4°C overnight. Cryostat sections (12 µm) were blocked using 3% BSA at room temperature for 30 minutes. For the flat-mount RPE immunostaining, the neural retinas were removed, and the rest of eye cups were fixed in methanol solution at 4°C for 10 minutes. For the flat-mount neural retina immunostaining, whole neural retinas were isolated in ice-cold DMEM solutions and fixed in 4% paraformaldehyde (Sigma-Aldrich) at 4°C for two hours. The flat mount RPE or neural retinas were blocked by a blocking buffer containing 3% bovine serum albumin (ST025; Beyotime Institute of Biotechnology) and 0.5% Triton X-100 (T8787; Sigma-Aldrich). Primary antibodies were used: anti-Rhodopsin (1:300, MAB5316; Millipore, Burlington, MA, USA), anti-M-Opsin (1:300, AB5745; Millipore), anti-S-opsin (1:200, sc-14363; Santa Cruz Biotechnology, Dallas, TX, USA) anti-OTX2 (1:300, AF1979; R&D Systems, Minneapolis, MN, USA), anti-ZO-1(1:100, 339100; Thermo Fisher Scientific, Waltham, MA, USA). The secondary antibodies (Alexa Fluor 488–or Alexa Fluor 594–conjugated donkey anti-rabbit or mouse immunoglobulin) were used at room temperature for two hours. DAPI (1:1000, C1002; Beyotime Institute of Biotechnology) was used to label the cell nuclei.

TUNEL staining was performed as previously described.^29^ Briefly, the paraffin retinal sections were dewaxed by xylene and boiled in sodium citrate (0.01 M; Beyotime Institute of Biotechnology) for two minutes. Then, sections were incubated with the TUNEL reaction Kit (Roche, 11684795910) according to the manufacturer's protocol.

### Transmission Electron Microscopy

The protocol for transmission electron microscopy (TEM) has been performed as previously described.^33^ Briefly, a mark was made at the 12 o'clock position of the cornea before the eyeball was dissected. The retinas were divided into dorsal and ventral parts, and the central dorsal and ventral patches (about 2 mm × 2 mm) were fixed in 2.5% glutaraldehyde for three hours at room temperature and then post-fixed in 1% osmium tetroxide for one hour at 37°C. After washing with PBS, the retinas were treated with 1% phosphotungstic acid and 1% sodium uranyl acetate for one hour at 37°C, dehydrated through acetone series and epoxy resin-acetone mixture at 37°C, and then embedded in epoxy resin at 45°C. Semi-thin sections were cut and stained with methylene blue (Sigma-Aldrich) to localize the RPE layer. Ultrathin sections were cut and mounted on grids, and the specimens were examined and photographed with a Hitachi H-7500 transmission electron microscope (Hitachi, Tokyo, Japan).

### Melanin Content Analysis

Melanin levels in RPE cells were measured as described previously.^33^ Briefly, dorsal and ventral eye cups were dissected in DMEM solution, and the neural retinas were removed. The rest of the eye cups were lysed with RIPA on ice for 10 minutes using a Micro Tissue Grinder and spun in a centrifuge at 10,000*g* for 10 minutes. The supernatant was used to measure protein concentration as an internal reference, and the pigmented precipitate was solubilized in 60 µL of 1 M NaOH and 10% dimethyl sulfoxide for two hours at 80°C. Solubilized melanin was assessed by absorbance at 405 nm.

### Intravitreal Injection

Mice were anesthetized by ketamine and xylazine, and the pupils were dilated by the 1% atropine eye drops. Approximately 0.5 µL SI (5 mg/mL) was introduced into the intravitreal center, which is just over the optic nerve head. After three days of SI injection, mice were subjected to retinal degeneration analysis.

### Statistical Analysis

Each experiment was repeated at least four times. Results are presented as mean ± SEM. Statistical analyses were performed using GraphPad Prism 8 (GraphPad, San Diego, CA, USA) with Student's *t*-test for comparing two groups, and one-way ANOVA with Bonferroni post-hoc test for comparing more than two groups. *P* < 0.05 was considered significant.

## Results

### Dorsal-Central RPE Is More Sensitive to SI Injury

**Figure 1.**
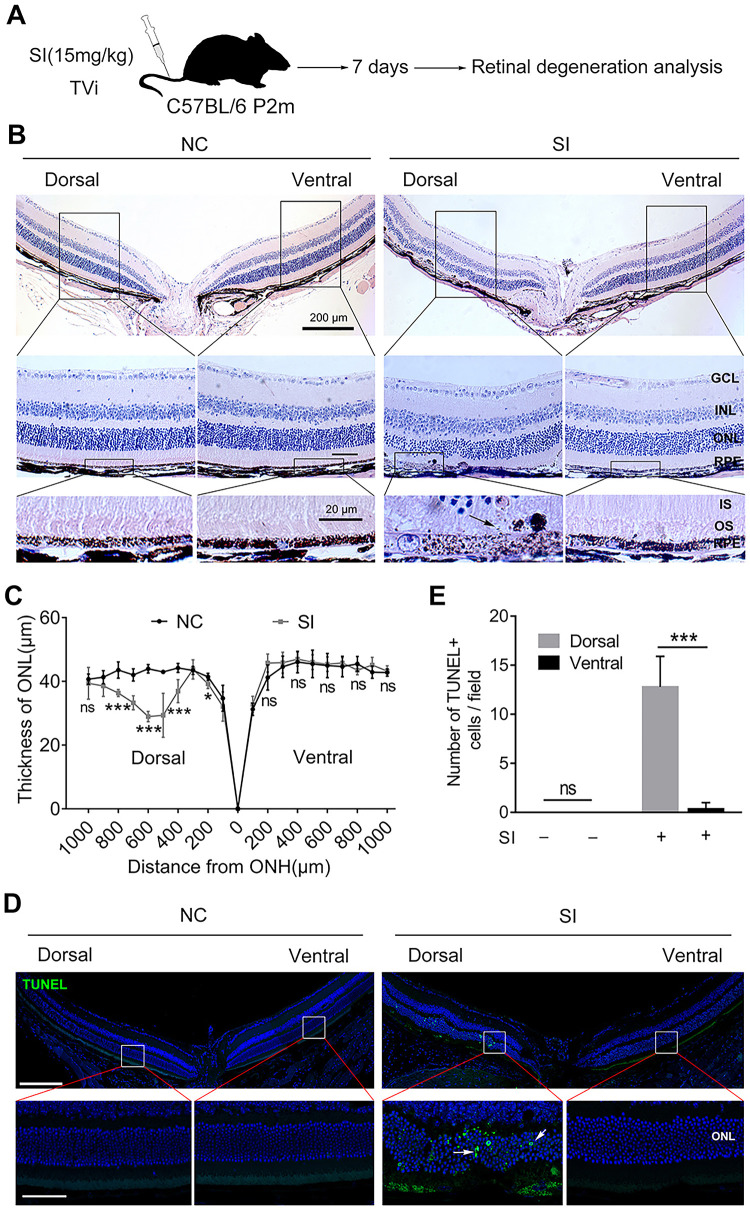
The dorsal retina exhibits severe damage in C57BL6/J mice treated with a low dose of SI. (**A**) Schematic representation of the experimental procedure of SI injury. A two-month-old C57BL/6 mouse was injected with a low dose of SI (15 mg/kg) via the tail vein, and retinal degeneration analysis was performed on day 7 after the single injection. (**B**) Histological structure of retinas from mice after seven days of low-dose SI or saline solution injection. The *solid black arrow* indicates RPE swelling and abnormal release of melanin granules apically into the photoreceptor outer segment. *Scale bar*, 200 µm (*upper panel*), 50 µm (*middle panel*), and 20 µm (*lower panel*). (**C**) Quantification of the thickness of the outer nuclear layer from the mice injected with a low dose of SI or saline solution. n = 6. (**D**) TUNEL assay of the retinas from the C57BL/6 mice in control and experimental groups. *Scale bar*, 200 µm (*upper panel*) and 50 µm (*lower panel*). (**E**) Quantification of the number of TUNEL-positive cells per field in the retina in the control and experimental group. n = 6. *P < 0.05, **P < 0.01, ***P < 0.001. Data are presented as the mean ± standard error of the mean and were compared using a Student *t*-test or one-way ANOVA when appropriate. GCL, ganglion cell layer; INL, inner nuclear layer; IS, photoreceptor inner segment; NC, Normal control; ONH, optic nerve head; ERG, electroretinography.

We first aimed to analyze whether and to what extent the dorsal or ventral RPE are prone to damage, considering the functional and morphological retinal asymmetry of the mouse.^19^ SI is widely used to induce a GA-like phenotype by causing RPE and secondary photoreceptor degeneration,^34^^,^^35^ thus mimicking the regional RPE degeneration in humans. Thus we used a low dose of SI (15 mg /kg) to induce RPE degeneration in adult C57BL/6 mice (Fig. 1A). Interestingly, H&E staining showed that the dorsal-central RPE was severely damaged by a low dose of SI, including signs of depigmentation and the release of melanin apically to the photoreceptor outer segment, whereas the ventral RPE still maintained a relatively integrated single layer at seven days after SI injection (Fig. 1B). In addition, TEM images further showed abnormal distribution of melanin granules in the dorsal outer segment (OS) and severe disruption of tight connections between adjacent dorsal RPE cells (Supplementary Fig. S1A). These data suggest that the dorsal central RPE is selectively damaged by the low dose of SI. It is well established that RPE damage often leads to secondary photoreceptor degeneration.^2^ Indeed, the thickness of the ONL overlying the dorsal damaged RPE was reduced significantly; however, the thickness of the ventral ONL was not notably altered by a low dose of SI (Figs. 1B, 1C). In addition, the TUNEL positive cells were almost exclusively restricted to the dorsal ONL (Figs. 1D, 1E). These data suggested that dorsal-central retinas, particularly the RPE and secondary photoreceptors, were significantly degenerated on SI treatment at 15 mg/kg.

**Figure 2.**
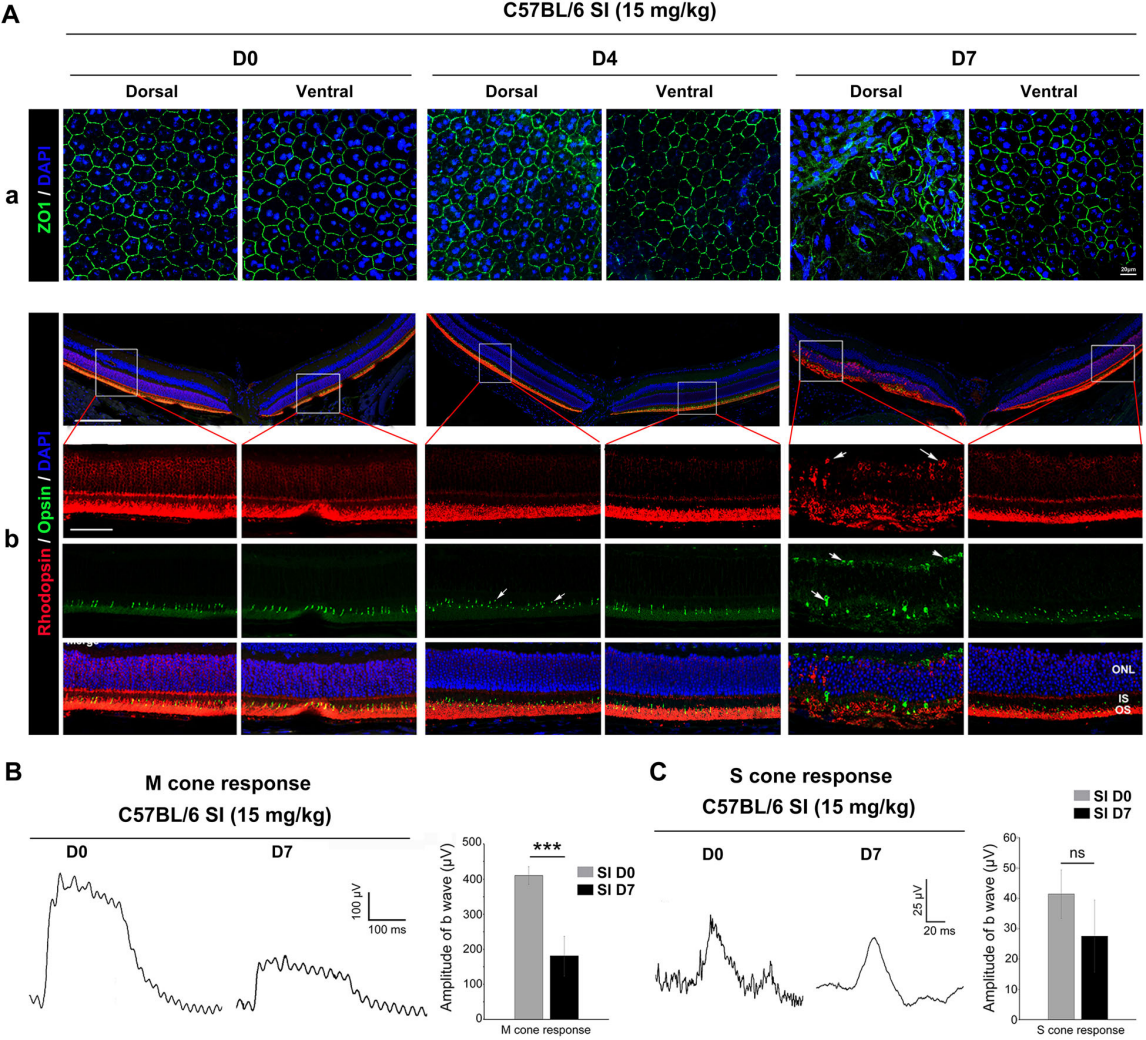
M cone degeneration was severe in C57BL6/J mice treated with a low dose of SI. (**A**) Immunostaining of anti-ZO1 (a), anti-Rhodopsin and Opsin (b) in the retinas treated with SI at day 0, day 4 or day 7. The solid white arrows indicate the incorrect localization of Rhodopsin or Opsin in the damaged dorsal retinas. ERG traces of the (**B**) M-cone and (**C**) S-cone responses from C57BL/6 mice injected with saline or low dose SI were elicited by green light with strength of 0.75 cd-s/m2 and UV light with strength of 3.00 mW-s/m2, respectively. The *right bar graph* shows the quantification of the amplitude of the b-wave from the (**B**) M-cone response and (**C**) S-cone response. n = 5.***P < 0.001. Data are presented as the mean ± standard error of the mean and were compared using a Student *t*-test. IS, photoreceptor inner segment; NC, Normal control.

To further analyze the retinal degeneration and damage extent of RPE and photoreceptors under low-dose SI treatment, we examined expression of zonula occludens-1 (ZO1), rhodopsin, and opsin in the SI-treated retinas. Immunostainings showed that hexagonal signal of ZO1 was present both in the dorsal and ventral RPE at day 4 after SI administration; however, it was present only in the ventral RPE at day 7 after SI administration (Fig. 2A, a). Interestingly, at day 4 after SI injection, an abnormal spot signal of M opsin was only observed in the dorsal retina, but rhodopsin was present in the OS of both the dorsal and ventral retinas (Fig. 2A, b). At day 7 after SI treatment, both rhodopsin and M opsin were mistrafficked toward the ONL of the dorsal damaged area instead of the ventral retina (Fig. 2A, b). These data suggested that RPE and photoreceptors were severely damaged in the dorsal retina at day 7 after SI treatment and that M cones were damaged before the RPE and rods. We further analyzed the cone and rod visual function by electroretinography, which showed that the amplitude of the b wave in the M cone response was significantly decreased at both day 4 and day 7 after SI injection (Supplementary Figs. S1B, S1C, and Fig 2B), whereas the amplitude of the b wave in the rod response was slightly decreased at day 4 after SI administration (Supplementary Figs. S1B, S1C). Furthermore, the amplitude of the b wave in the S cone response was not significantly different after SI administration, even seven days afterward (Fig. 1H). These results suggest that M cones are differentially damaged by a low dose of SI.

**Figure 3.**
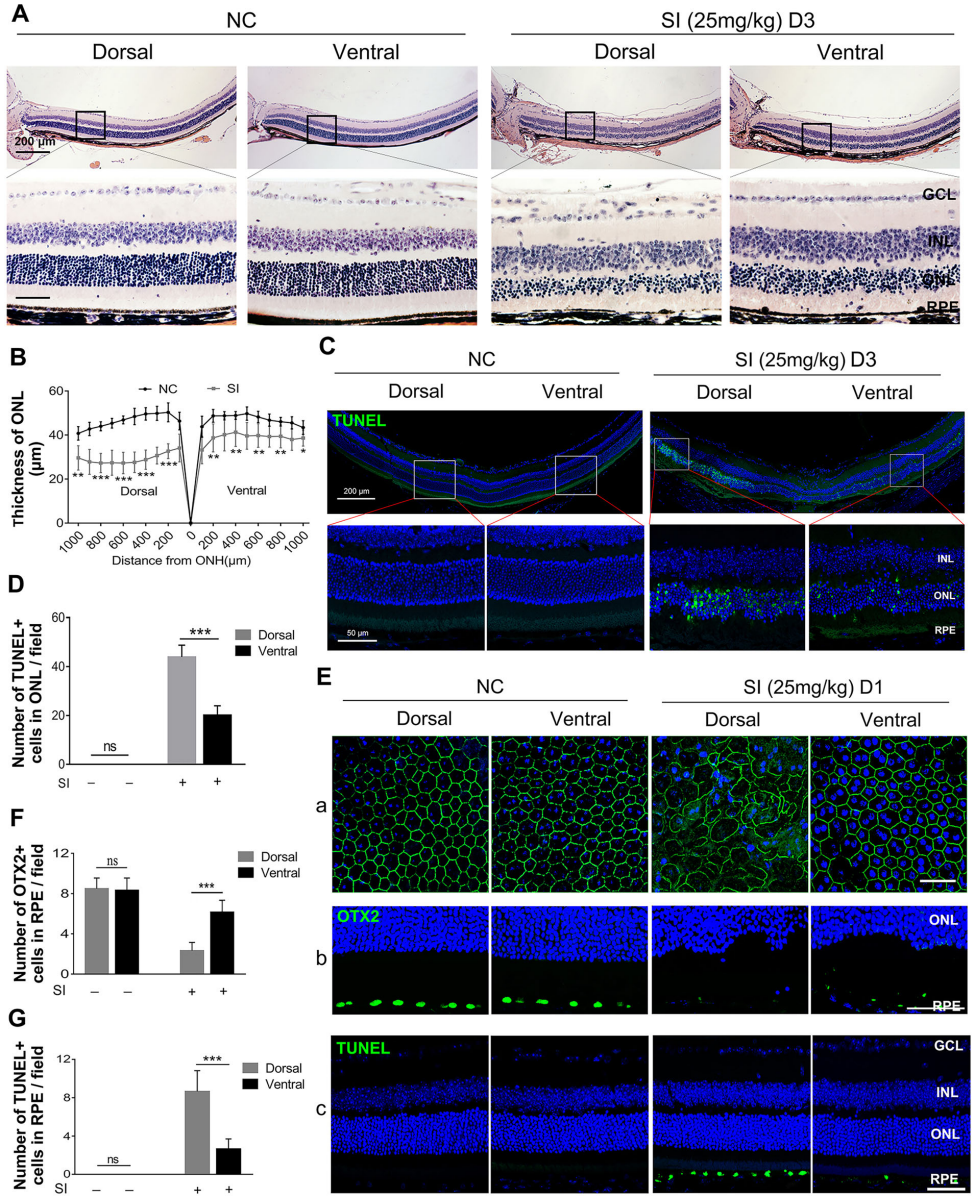
High dose of SI treatment induces severe retinal degeneration in the dorsal visual field of C57BL/6 mice. (**A**) Histological images of H&E staining from two-month-old C57BL/6 retinas treated with saline solution (*left panels*) or high-dose SI (*right panels*) (25 mg/kg) after three days. *Scale bar:* 200 µm (*upper panel*), 50 µm (*lower panel*). (**B**) Quantification of the thickness of the ONL from C57BL/6 retinas treated with saline solution or a high dose of SI. (**C**) Images of TUNEL assays from the C57BL/6 retinas three days after intraocular injection of saline or a high dose of SI and (**D**) quantitative analysis of the cell death rate of photoreceptor cells. (**E**) RPE degeneration analysis based on (**a**) ZO1 staining, (**b**) OTX2 staining, and (**c**) TUNEL detection of C57BL/6 mice one day after treatment with a high dose of SI. (**F**, **G**) Quantification of the number of (**D**) OTX2 D) and (**E**) TUNEL positive cells in the RPE. n = 6. *P < 0.05, **P < 0.01, ***P < 0.001. Data are presented as the mean ± standard error of the mean and were compared using a one-way ANOVA. GCL, ganglion cell layer; INL, inner nuclear layer; IS, photoreceptor inner segment; NC, Normal control; ONH, optic nerve head.

To investigate the different responses of dorsal and ventral RPE to a high dose of SI, we injected 25 mg/kg of SI (high-dose) into adult C57BL/6 mice and analyzed the resultant retinal degeneration. After three days of a single SI injection, histological images showed that both dorsal and ventral RPE were significantly degenerated, but the dorsal RPE showed a large area of cell loss and was more markedly damaged than the ventral RPE, which maintained a relatively complete epithelial layer (Fig. 3A). As expected, the secondary photoreceptor degeneration was more severe in the dorsal than in the ventral retina, showing considerable thinning of the dorsal ONL (Figs. 3A, 3B), as well as a large number of TUNEL-positive cells (Figs. 3C, 3D). To further investigate the damage of the RPE, we analyzed RPE degeneration at one day after SI injection at a high dose. The immunohistochemical expression profile of ZO1, a specific membrane protein of RPE, showed that the hexagonal structure of dorsal RPE disappeared completely after SI injection, whereas a larger part of the ventral RPE maintained a normal structure (Fig. 3Ea). In addition, the expression of OTX2, a specific marker of RPE, was more markedly decreased in the dorsal than in the ventral RPE (Figs. 3Eb, 3F). Furthermore, TUNEL data also showed that the number of TUNEL-positive cells was higher in the dorsal than in the ventral RPE (Figs. 3Ec, 3G). Together, these data further indicate that the dorsal RPE is more sensitive to SI injury than the ventral RPE.

To circumvent the effect of tail vein injection on the sensitivity of dorsal-ventral RPE to SI injury, SI (2.5 µg) was intravitreally injected into the eyes of two-month-old C57BL/6 mice (Supplementary Fig. S2A). At three days after SI injection, large vacuoles were observed in the dorsal-central RPE, whereas the ventral RPE still maintained a relatively intact single layer (Supplementary Fig. S2B). Besides, the number of OTX2-positive cells was evidently decreased in the dorsal RPE compared with the ventral RPE after SI treatment (Supplementary Figs. S2C, S2D). These data indicate that dorsal-central RPE was more severely damaged than the ventral RPE after SI injury by intravitreal injection, suggesting that dorsal-central RPE is indeed differentially sensitive to SI injury.

### RPE Shows Dorsal-Ventral Gradient in Pigmentation

**Figure 4.**
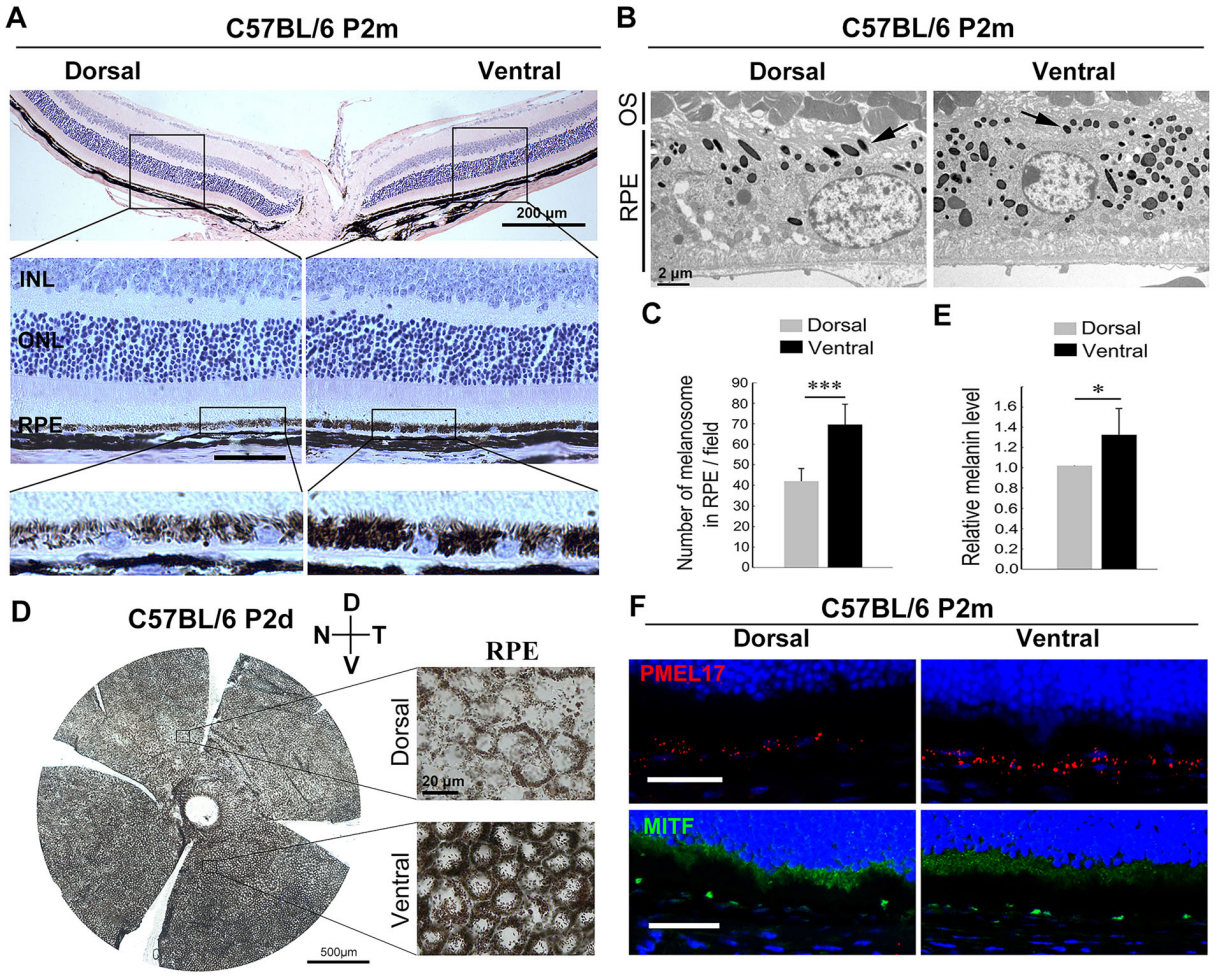
Different levels of pigmentation between the dorsal and ventral RPE. (**A**) Representative H&E staining image of sectional retina from two-month-old C57BL/6 mice kept under normal conditions (n = 8). Scale bars, 200 µm (*upper* panel) and 50 µm (*lower panel*). (**B**) Melanosomes in the dorsal and ventral RPE cells were revealed by TEM. The *black arrows* indicated the melanosomes. *Scale bars:* 2 µm. (**C**) Quantification of melanosomes in the RPE cells based on the TEM analysis (n = 6). ***P < 0.001 by Student's *t* test. (**D, E**) Images of the flat-mounted RPE of the C57BL/6 mice at postnatal day 2 (**D**) and the quantification of melanin content of the dorsal and ventral RPE (**E**) (n = 4). *Scale bars:* 500 µm (flat-mounted RPE) and 20 µm (*right enlarged images*). **(F)** Representative images of immunostaining of dorsal and ventral RPE from two-month-old C57BL/6 mice detected by anti-PEML17 and anti-MITF antibodies (n = 5). Data are presented as the mean ± standard error of the mean, and were compared using Student's *t*-test. *P < 0.05, ***P < 0.001. INL, inner nuclear layer; D, dorsal; V, ventral; N, nasal; T, temporal.

The different responses of RPE regions to SI injury prompted us to investigate the different features between these regions, the dorsal and ventral RPE. Interestingly, histological images showed a difference in density of pigmentation between dorsal and ventral RPE in normal conditions. The dorsal RPE was hypopigmented, whereas the ventral RPE was hyperpigmented (Fig. 4A). Consistently, the number of melanosomes in the dorsal RPE was lower than in the ventral RPE (Figs. 4B, 4C), indicating that the RPE bears a dorsoventral gradient of pigmentation. To further confirm this, we analyzed RPE pigmentation using whole flat-mount RPE. However, because of the pigmented melanocytes in the underlying choroid, it was impossible to directly distinguish the difference in pigmentation between dorsal and ventral RPE in the flat-mount RPE. To address this issue, we instead analyzed the whole flat-mount RPE of neonatal C57BL/6 mice, at postnatal day 2 (P2), when the melanocytes were not pigmented in the choroid. Here, the melanosomes of RPE could be clearly observed, as well as the melanin content, which was lower in the dorsal than in the ventral RPE cells (Figs. 4D, 4E). Together, these results consistently indicate that RPE displays a pronounced pigmentation gradient along the retina dorsal-ventral axis.

Because of the high melanin content of ventral RPE, we further analyzed whether melanin synthesis-related genes, including, microphthalmia-associated transcription factor, (*Mitf*), Tyrosinase (*Tyr*) and pre-melanosomal protein (*Pmel17*), showed a higher expression in the ventral RPE. The results obtained by real time qPCR showed that most of these genes, such as *Mitf*, a critical regulator of RPE and melanocyte development,^36^ were equally expressed in the dorsal and ventral RPE. However, *Pmel17* expression was higher in the ventral RPE, according to the qPCR results (Supplementary Fig. S3A) and immunostaining images (Fig. 4F). Concerning MITF protein expression, Western blot results further showed that levels of MITF were equivalent in dorsal and ventral RPE (Supplementary Fig. S3B). Consistent with TEM results, these data further indicate that hyperpigmentation of ventral RPE results from a higher number of melanized melanosomes.

### Photoreceptor Degeneration Instead of RPE Degeneration Is Prevented in the Albino Mice Under a High Dose of SI Injury

**Figure 5.**
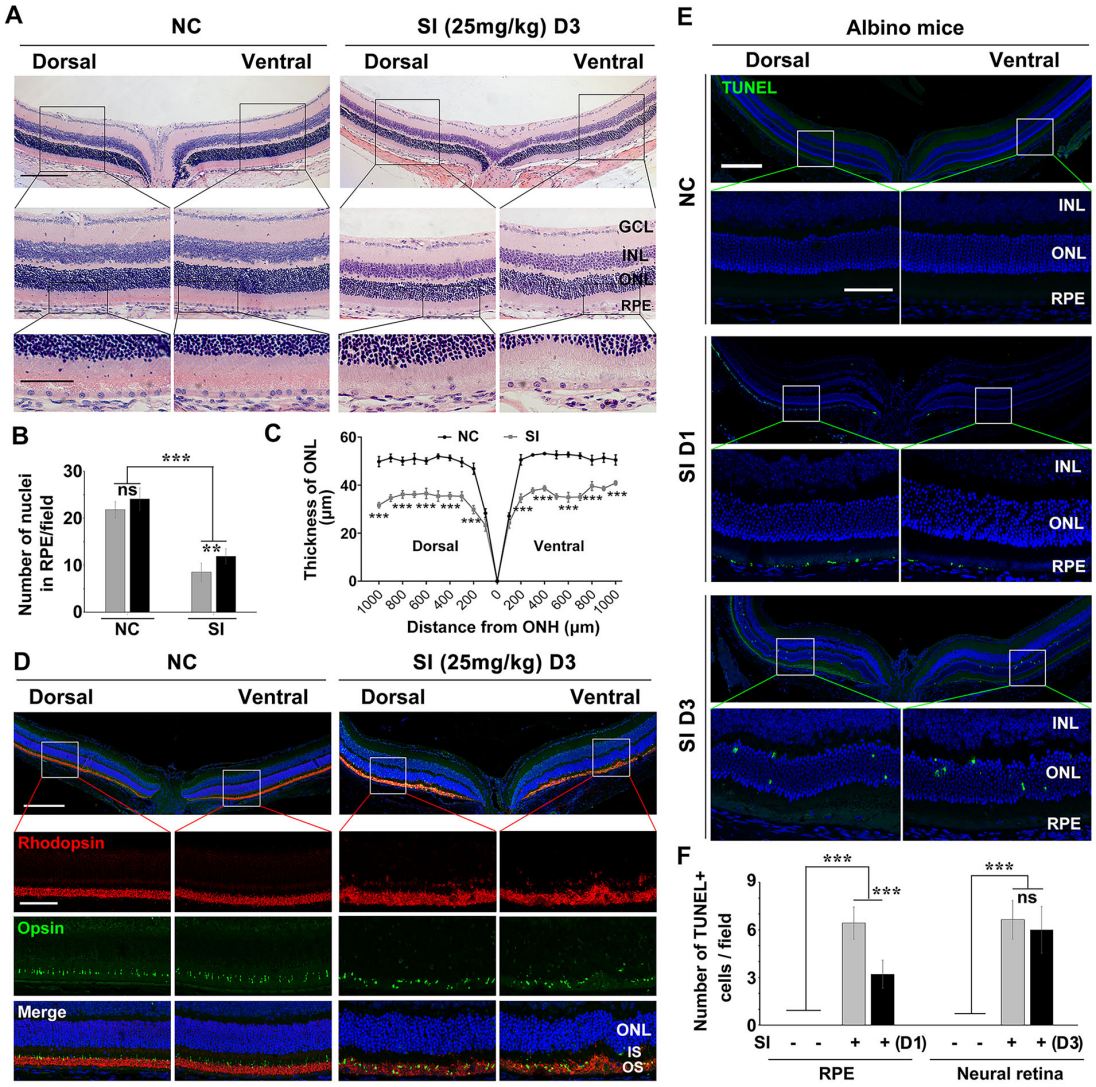
Deficiency of melanin granules does not accelerate SI-induced RPE degeneration. (**A**) Histological images of H&E staining from two-month-old albino mice three days after a single injection of saline solution or a high dose of SI (25 mg/kg). *Scale bar:* 200 µm (*upper panel*) and 50 µm (*lower panel*). (**B**) Quantification of the nuclei in the RPE and (**C**) the thickness of ONL from the albino mice under the indicated conditions. (**D**) Retinal degeneration analysis of the albino mice injected with high-dose SI by double staining of anti-Rhodopsin and Opsin. (**E**) Images of TUNEL assays from the two-month-old albino retinas treated with high-dose SI (25 mg/kg) for one or three days. *Scale bar:* 200 µm (*upper panel*), 50 µm (*lower pane*). (**F**) Quantitative analysis of the cell death rate in RPE (*left bars*) one day after injection of high dose SI and in the ONL (*right bars*) three days after injection of high dose SI (n = 6). *P < 0.05, **P < 0.01, ***P < 0.001. Data are presented as the mean ± standard error of the mean and were compared using a one-way ANOVA. NC, normal control; GCL, ganglion cell layer; INL, inner nuclear layer.

Given that melanin granules are hypothesized to have an antioxidant role in RPE,^37^ we assumed that the higher sensitivity of dorsal RPE to SI should be caused by a lower content of melanized granules. To address this question, we used adult albino mice (*Tyr^c^/Tyr^c^*), which showed normal RPE specification, but a defect in melanin granules in the nonpigmented RPE,^38^ to analyze the role of melanized granules in the response to SI. At three days after a high-dose SI (25 mg/kg) injection, histological images showed that the dorsal RPE appeared fractured, whereas the ventral RPE still maintained a relatively intact epithelial layer, although with a slight swelling (Fig. 5A). Furthermore, a fewer number of nuclei was found in the dorsal RPE than in the ventral RPE (Fig. 5B). These results indicate that the dorsal RPE is still more sensitive to SI injury in albino mice under a high dose of SI.

Next, we analyzed the secondary photoreceptor degeneration in albino mice treated with SI. Surprisingly, at three days after SI injection, the histological images showed that the thickness of the ONL was reduced to a similar degree between the dorsal and ventral retinas (Figs. 5A, 5C). In addition, immunostaining showed that, in albino mice at three days after SI injection, M opsin was slightly mislocalized in the ONL of the dorsal and ventral retinas, and Rhodopsin was primarily present in the OS of the dorsal and ventral retinas. These data suggest that photoreceptor degeneration is lower in albino mice than in C57BL/6 mice at three days after a high dose of SI (Figs. 5A, 5B).

To further confirm the difference in sensitivity between dorsal and ventral RPE and photoreceptors to SI injury in albino mice, we analyzed cell death at two timepoints after SI. As shown in Figures 5E and 5F, at one day after SI, the number of TUNEL-positive cells in the ventral RPE was considerably lower than in the dorsal RPE, similar to what observed in C57BL/6 mice (Figs. 3C, 3E). However, at three days after SI administration, the extent of cell death in dorsal ONL was similar to that in the ventral ONL in albino mice (Figs. 4E, 4F). Thus the number of dead cells in the dorsal ONL of albino mice was significantly lower (Figs. 5E, 5F) than C57BL/6 mice (Figs. 3C, 3D). These data further showed that photoreceptor degeneration was lower in albino mice than in C57BL/6 mice after a high dose of SI.

Collectively, these data indicate that the dorsal RPE degeneration is still more severe than the ventral RPE in albino mice on a high dose of SI; however, the sensitivity of dorsal photoreceptors to SI is lower in albino mice.

### RPE Degeneration Is Prevented in Albino Mice When Treated With a Low Dose of SI

**Figure 6.**
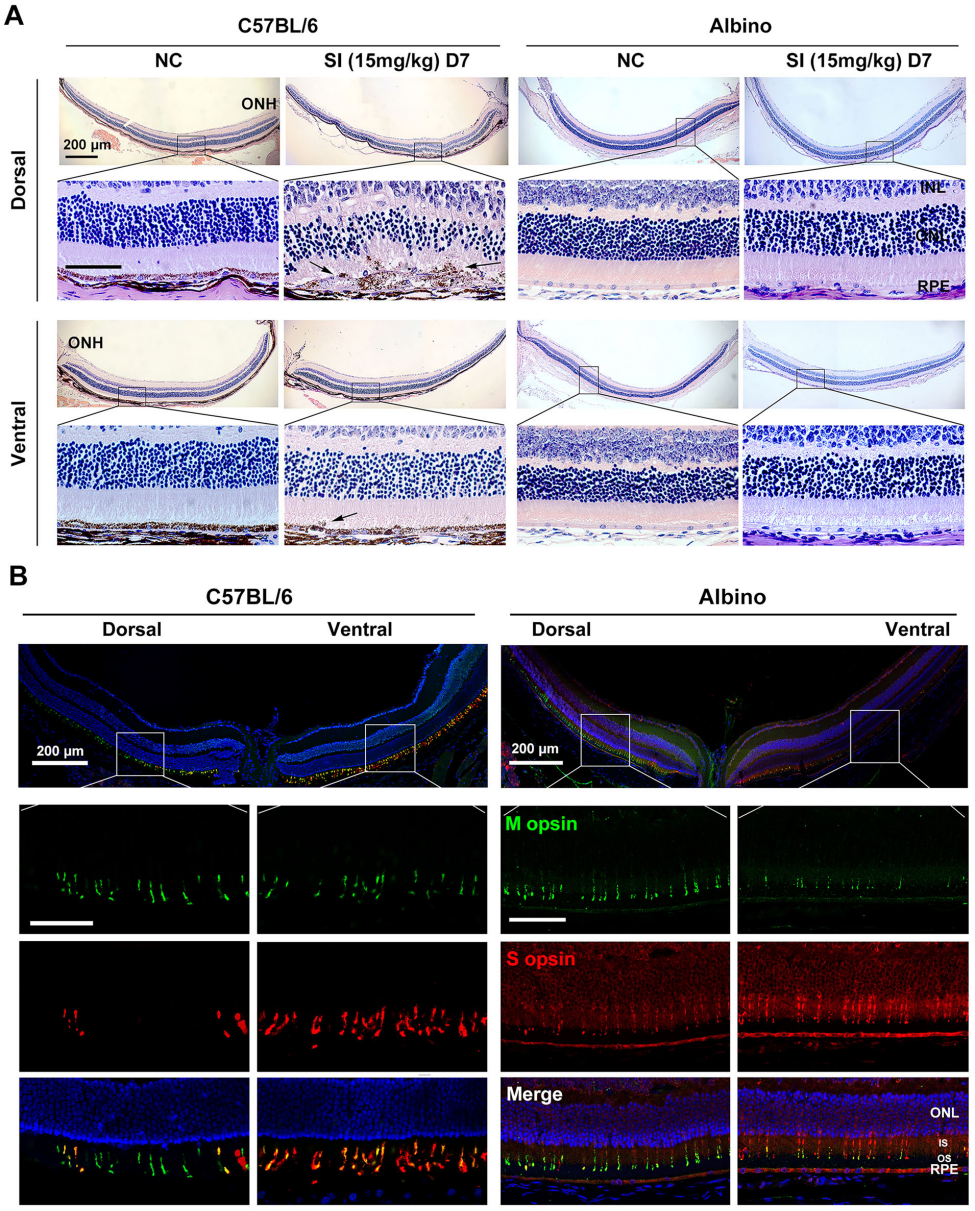
RPE degeneration is prevented in the dorsal retina of albino mice under low dose of SI treatment. (**A**) Histological images of H&E staining from two-month-old C57BL/6 (*left panels*) and albino (*right panels*) mice seven days after a single injection of saline solution or low dose of SI (15 mg/kg) (n = 6). The *black arrows* point to the mislocalization of melanin granules in the OS of dorsal retinas from the C57BL/6 mice. *Scale bar:* 200 µm (*upper panel*) and 50 µm (*lower panel*). (**B**) Immunostaining images of M and S opsins across the dorsoventral retinas of the C57BL/6 (*left panels*) and albino (*right panels*) mice by retinal sections (n = 6). INL, inner nuclear layer; IS, photoreceptor inner segment. *Scale bar:* 200 µm (*upper panel*) and 50 µm (*lower panel*).

To further analyze the sensitivity of albino RPE to different doses of SI injury, we injected them with a low dose of SI (15 mg/kg). At 7 days after a single SI injection, histological images showed that the ventral RPE still maintained an intact layer both in C57BL/6 and albino mice under a low dose of SI. Surprisingly, the dorsal central RPE maintained a relatively integrated layer even though it showed a slight swelling in the albino mice, while the dorsal central RPE in C57BL/6 mice showed evident swelling, abnormal release of melanin granules and sub-RPE deposits as above mentioned (Fig. 6A). In addition, the hexagonal signal of ZO1 was conserved in the dorsal and ventral RPE of albino mice at day 7 after SI treatment (Supplementary Fig. S4A). These data indicate that the extent of dorsal RPE damage was lower in albino mice than in C57BL/6 mice after a low dose of SI, suggesting that dorsal RPE degeneration was prevented in albino mice in these conditions.

Given that photoreceptors have been reported to be more sensitive to low doses of SI than RPE^39^ and that M cones, dominant in the dorsal retina, are the most sensitive to SI injury among photoreceptors, we decided to assess the role of M cones in RPE degeneration induced by a low dose of SI. It is well known that in mice, cone photoreceptors are classified in three types according to the expression pattern of opsin pigment,^40^ such as M cone (M opsin only), true S cone (S opsin only), and mixed cone (both M and S opsins).^41^ Currently, M cones have been reported to be replaced by mixed cones in the dorsal retinas of albino mice.^23^ Indeed, we confirmed these results by analyzing the distribution of M cones and mixed cones in the dorsal and ventral retina of albino mice using immunostaining. These data consistently support previous results showing that mixed cones are dominant in the albino dorsal retina (Fig. 6B). However, cone photoreceptors still showed a pronounced gradient in opsins along the dorsal-ventral axis. In addition, in albino mice, M opsin was highly expressed in the dorsal central retina, whereas S opsin was highly expressed in the ventral retina (Fig. 6B). Furthermore, the amplitude of the b wave in M cone response and rod response were not altered in albino mice at day 7 after SI treatment (Supplementary Figs. S4B, S4C). Collectively, these data indicate that dorsal RPE degeneration is prevented in albino mice with dominant mixed cones in the dorsal retina, which suggests that dorsal RPE degeneration in C57BL/6 mice under a low dose of SI is associated with the overlying M cones.

### SI-Induced RPE Degeneration Is Prevented in 129 Mice with Predominant Mixed Cones in the Dorsal Retina

**Figure 7.**
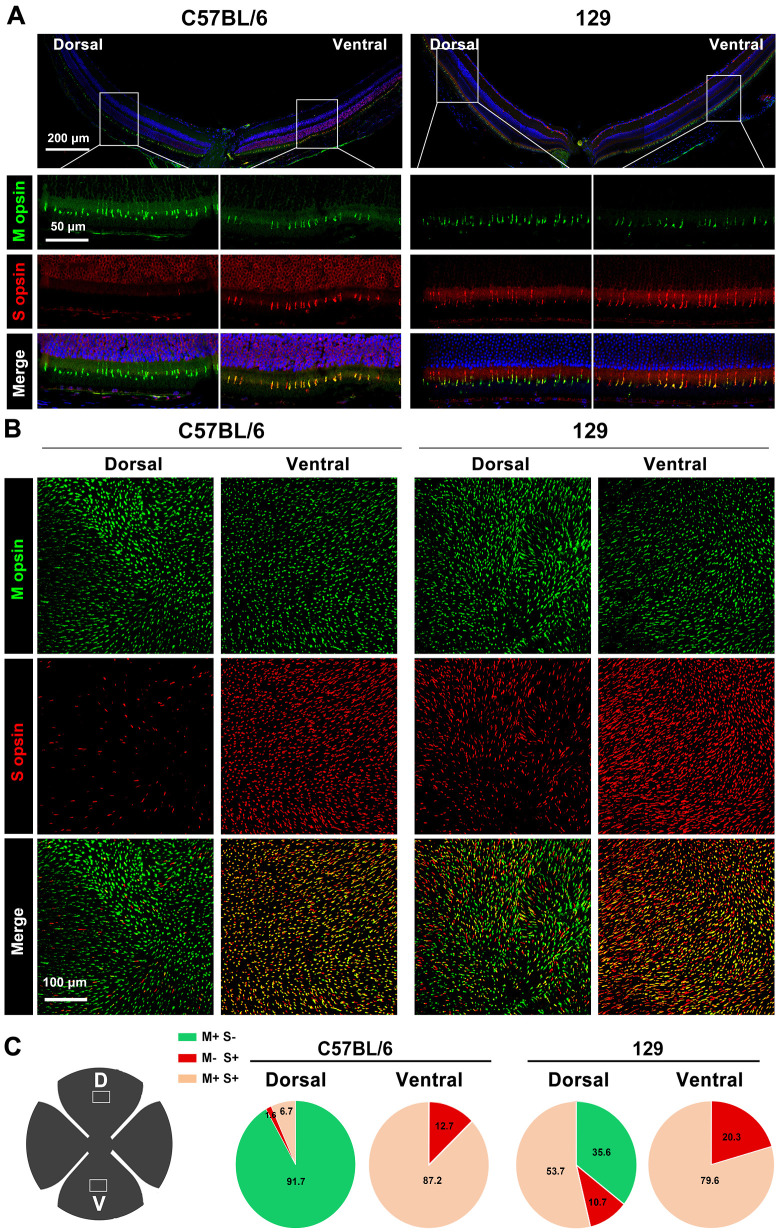
The dorsal retina is rich in mixed cones instead of M cones in 129 mice. (**A, B**) Immunodetection of M and S wavelength-sensitive opsins in (**A**) dorsal and ventral retinas from two-month-old C57BL/6 (*left panels*) and 129 (*right panels*) mice by retinal sections and (**B**) flat-mounted retinas. (**C**) The *white square* in the retinal scheme shows the analyzed dorsal and ventral regions for detecting M and S opsins, and the *pie graphs* show the percentage of cones manually classified as M+S− (*green*), S+M− (*red*), and M+S+ (*mixed, pink*) based on the opsin expression in the dorsal and ventral retinas from these two strain mice (n = 4). D, dorsal visual field; V, ventral visual field. M, M-opsin; S, S-opsin.

To further analyze the role of M cones in SI injury, we attempted to find a mouse model showing deficiency of M cones in the dorsal central retina. Interestingly, the mice on the 129 genetic background showed a dominance of mixed cones in the dorsal retina as well. Immunostaining images in C57BL/6 mice showed that only M opsin was observed in the dorsal retina, whereas M and S opsin types were overlapped in the ventral retina, as previously reported (Fig. 7A).^23^ However, in the 129 mice, the majority of the M opsins were overlapped by S opsins in both the dorsal and ventral retina (Fig. 7A). To confirm this result, immunostaining of flat-mount retinas was performed. As shown in Figure 7B, M opsin was extensively overlapped by S opsin both in the dorsal and ventral retina in 129 mice. However, in C57BL/6 mice, M opsin was densely distributed in the dorsal central retina and overlapped by S opsin in the ventral retina (Fig. 7C). These results consistently indicated that the dorsal-central dominant M cones were replaced by mixed cones in 129 mice.

**Figure 8.**
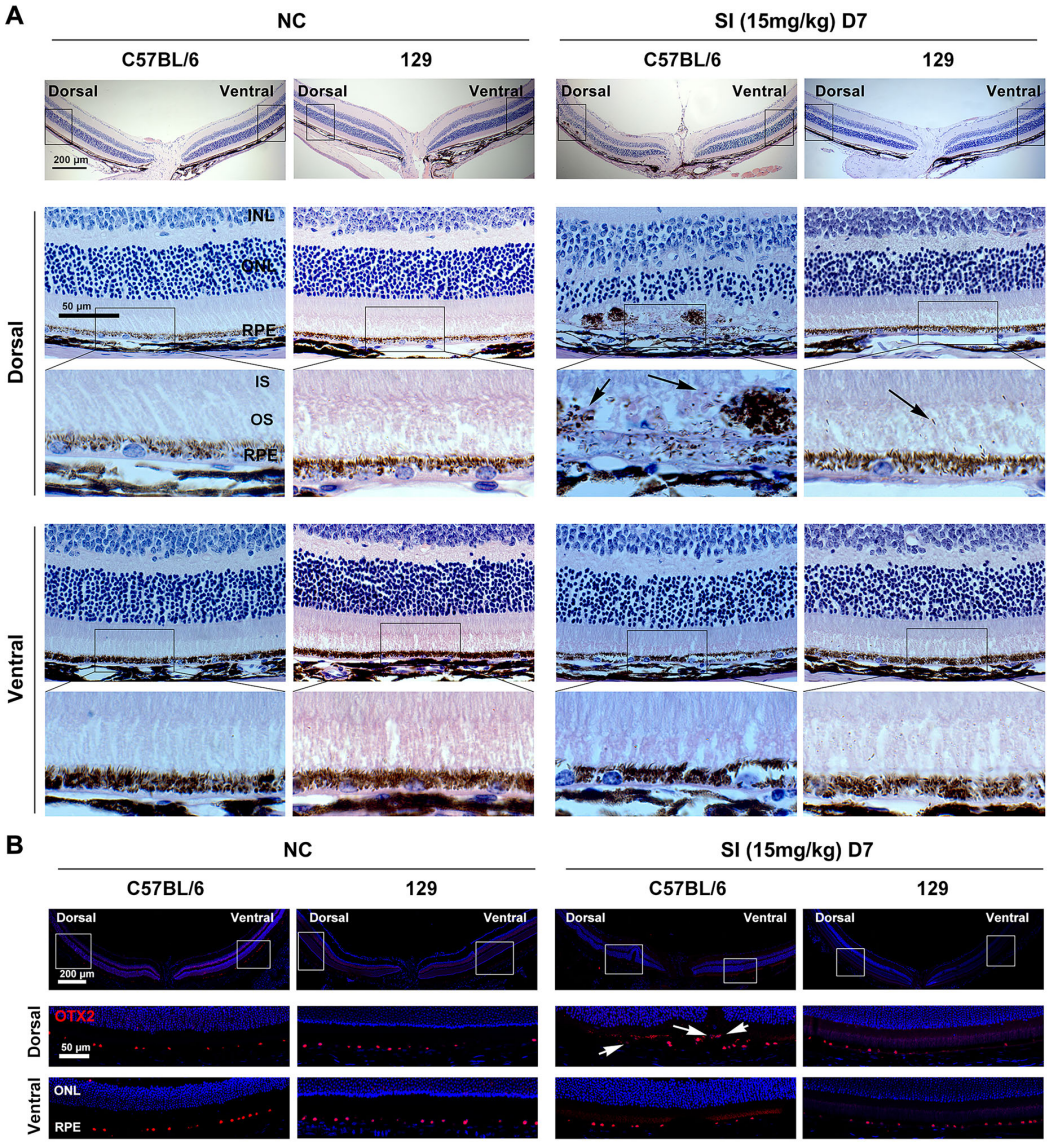
RPE degeneration in the dorsal region is prevented in 129 mice treated with a low dose of SI treatment. (**A**) Histological images of H&E staining from two-month-old C57BL/6 (*left panels*) and 129 (*right panels*) mice seven days after a single injection of saline solution or low dose of SI (15 mg/kg) (n = 6). The *black arrows* point to the mislocalization of melanin granules in the OS of dorsal retinas from the C57BL/6 and 129 mice. *Scale bar:* 200 µm (*upper panel*) and 50 µm (*lower panel*). (**B**) Immunodetection of OTX2 in the RPE of C57BL/6 (*left panels*) and 129 (*right panels*) mice under the indicated conditions (n = 6). The *white arrows* point to the abnormal accumulation of RPE cells in the dorsal subretinal cavity of C57BL/6 mice under low dose of SI treatment. *Scale bar:* 200 µm (*upper panel*) and 50 µm (*lower panel*). INL, inner nuclear layer; IS, photoreceptor inner segment.

We next analyzed retinal degeneration of 129 mice under a low dose of SI (15 mg/kg). Histological images showed that, in normal conditions, RPE of 129 mice showed a pronounced pigmentation gradient along the dorsal-ventral axis, similar to C57BL/6 mice (Fig. 8A). However, the response of dorsal RPE to SI injury differed significantly between these two strains of mice. As shown in Figure 7A, at seven days after SI injection, the layer of dorsal-ventral RPE showed severe degeneration and subretinal pigmented cell deposits in C57BL/6 mice, whereas it was still intact in the 129 mice, even though incorrect localization of melanin granules was observed in the dorsal OS. To analyze whether the pigmented cells deposited in the subretina of C57BL/6 mice were RPE cells, we examined OTX2 expression in the retinas of the two mouse strains. As shown in Figure 8B, OTX2-positive cells were abnormally deposited in the dorsal subretina of the C57BL/6 mice after SI injury, suggesting that the pigmented cells were indeed damaged RPE cells in these mice. On the contrary, OTX2 expression after SI injury was normal in the ventral RPE of C57BL/6 mice, as well as in both the ventral and dorsal RPE of 129 mice. Furthermore, the hexagonal structure of RPE was maintained in the dorsal and ventral retinas of the 129 mice at day 7 after SI treatment (Supplementary Fig. S5A). These data indicate that dorsal RPE degeneration in 129 mice is slighter than in C57BL/6 mice under a low dose of SI. However, the amplitude of the b wave in M cone response was dramatically decreased in 129 mice at day 7 after SI treatment; however, that of the rod response was slightly decreased, suggesting that the retinal function was also disrupted in 129 mice after SI treatment (Supplementary Figs. S5B, S5C).

Collectively, these data indicate that, under a low dose of SI, dorsal RPE degeneration is significantly attenuated in 129 mice possessing dominant mixed cones in the dorsal central retina. This suggests that the overlying M cones contribute to the observed dorsal RPE degeneration in C57BL/6 mice on a low dose of SI.

## Discussion

**Figure 9.**
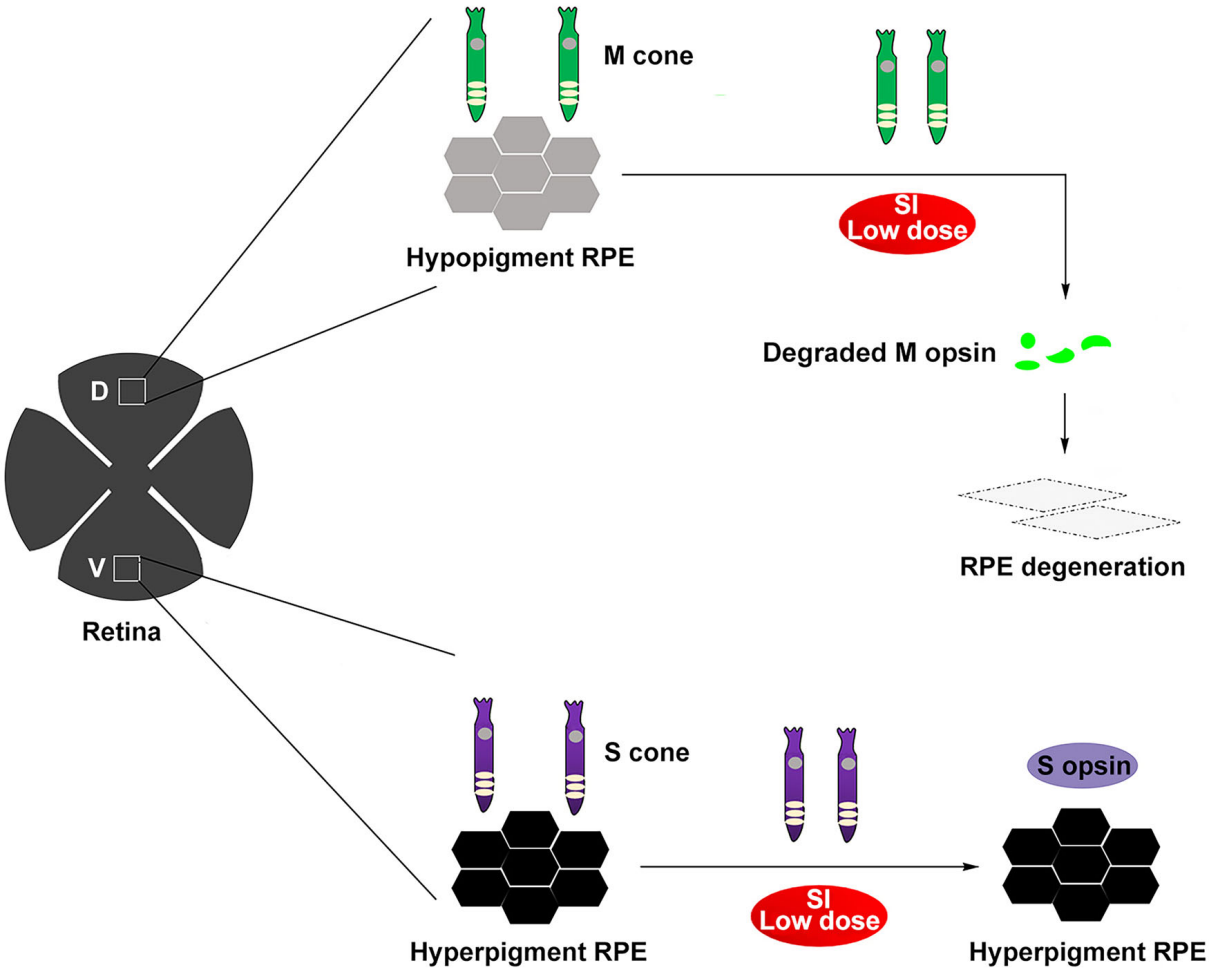
A potential mechanism of selective dorsal RPE degeneration in C57BL/6 mice under low dose SI treatment. The dorsal RPE is hypopigmented and is predominantly covered by M cones characterized by expression of M opsin, and the ventral RPE is hyperpigmented and predominantly covered by S cones characterized by expression of S opsin. M opsin is thought to be more unstable than S opsin under a cyclic light condition, thus M cones are much more sensitive to oxidative stress than S cones, such as that caused by SI injury. Under low dose SI treatment, M opsin was notably degraded in the dorsal M cones, and the damaged M cones likely induced the dorsal RPE degeneration.

RPE has been so far thought to consist of homogeneous cells supporting the overlying photoreceptors.^1^ The present study showed that RPE cells were heterogenous in pigmentation along the dorsal-ventral axis, showing selectively dorsal RPE degeneration in a SI-induced retinal degeneration model in mice. Unexpectedly, the extent of regional RPE degeneration could be regulated by the overlaying M cones rather than melanin granules as schematically illustrated in Figure 9. Collectively, our findings support a novel concept in which photoreceptors are not only affected by RPE dysfunction but also adversely regulate RPE degeneration, suggesting that M cones could serve as a novel potential target for the treatment of RPE degeneration.

The effect of the RPE on photoreceptor degeneration is well-established. Conversely, the effect of photoreceptors on RPE degeneration remains partially understood. It has been shown that loss of photoreceptors leads to RPE morphological remodeling,^42^ suggesting that photoreceptors do affect RPE cells under certain conditions. Here, we found that RPE degeneration was associated with overlying M cones in the SI-induced retinal damage model. This sensitivity of the dorsal-central RPE to SI injury could be modified through the distribution of M cones, as in albino or 129 mice, SI-induced RPE degeneration was prevented in the dorsal retina, primarily composed of mixed cones. At the molecular level, M opsin protein in M cones might mediate the higher sensitivity of dorsal-central RPE to SI injury. It has been shown that M opsin is an unstable protein, prone to being expressed in a truncated form or mistrafficked into the ONL under stress conditions,^43^ and that M opsin deletion prevents M cone degeneration in a retinal degenerative disease model.^44^ Therefore, high levels of M opsin may underlie an increased risk to M cone and dorsal RPE degeneration in the SI injury model. Of note, this risk seems to be reduced by expression of S opsin, because expression of both S and M opsins in the mixed cones allowed the preservation of the dorsal RPE in 129 and albino mice upon a low dose of SI. This could be due to a compensation of M opsin function by S opsin in the mixed cones in a manner similar to the way M opsin compensates for Rhodopsin to rescue rod function and degeneration in *Rho* mutant mice.^45^ Taken together, our findings provide a potential regulatory network in which RPE degeneration is somehow triggered by M cones in SI-induced injury. In this sense, it will be important to clarify the detailed mechanisms of how M cones regulate RPE degeneration in the future.

Intriguingly, although RPE degeneration was present in albino mice, compared with that in C57BL/6 mice, under low-dose SI treatment, during high-dose SI injury, RPE degeneration in albino mice was similar to that in the C57BL/6 mice as previously reported.^46^ This phenomenon could be attributed to differences in the action manner of SI at low and high doses. It is well established that cell death is typically first observed in the RPE, not in the ONL, on a high dose of SI.^35^ Conversely, photoreceptor degeneration occurs before RPE damage under a low dose of SI,^39^ particularly degeneration of M cones, which are the most sensitive to SI injury among the photoreceptor cell types. Therefore a high dose of SI could directly target RPE to induce secondary photoreceptor degeneration, whereas a low dose of SI may directly target photoreceptor cells and induce RPE degeneration afterward. Thus RPE damage might be independent of the overlying M cones under high-dose SI treatment. However, it is still unclear why the RPE damage is more severe at the dorsal than at the ventral area on a high dose of SI.

After RPE degeneration, secondary photoreceptor degeneration is thought to be caused by the RPE dysfunction, such as failures in regeneration of visual cycle pigments, support of trophic factors, and phagocytosis of spent photoreceptor outer segments.^1^ In the present study, we found that secondary photoreceptor degeneration differed between C57BL/6 and albino mice under a high dose of SI. There are two explanations for this difference. On one hand, the severe photoreceptor damage in dorsal retinas of C57BL/6 mice could be caused by the dominantly M cones, because M cones are very sensitive to SI injury. In albino mice, dorsal mixed cones are present instead of M cones, and that confers a low sensitivity to SI. This results in a considerably lower photoreceptor damage in the dorsal retinas of albino mice during SI injury. On the other hand, the abnormal release of melanin granules by the damaged RPE could also contribute to photoreceptor degeneration in C57BL/6 mice. Melanized melanosomes are consistently localized in the apical region of the RPE^47^ and usually thought to play antioxidant roles to avoid photo-oxidative stress.^48^ However, melanosomes have also been shown to damage RPE after modification by lipofuscin.^49^ We found that melanin granules were abnormally released from SI-damaged RPE and apically toward OS in the dorsal retinas of C57BL/6 mice. The melanin granules are supposed to be cleared by macrophages or Müller glial cells,^35^ both of which can secrete cytokines to induce inflammation during retinal degeneration.^50^ Therefore melanin granules in the OS probably trigger an inflammatory response to accelerate photoreceptor degeneration during SI injury, and thus the defect of melanin granules could prevent dorsal photoreceptor degeneration in albino mice under a high dose of SI.

In addition to the different sensitivities between the dorsal and ventral RPE to SI injury, we also found heterogeneity in RPE cell pigmentation along the dorsal-ventral axis. Although RPE had been considered as a homogenous epithelium in mice, the inherent variability of RPE cells has been gradually revealed. In mature mouse retinas, certain heterogeneous features are observed between the central and peripheral RPE, such as cell size and orientation.^24^ However, according to the visual function, the mouse retina is divided into dorsal and ventral fields, unlike human retina with central and peripheral visual fields. Here, we found that RPE shows a pronounced pigmentation gradient between dorsal and ventral regions, which is accompanied by the dorsoventral gradient in opsin expression across the cone photoreceptors. Intriguingly, the difference of RPE pigmentation along the dorsal-ventral axis could provide optimal conditions for normal function of cones, such as avoiding absorption of excessive light. Indeed, the ventral hyperpigmented RPE can absorb considerably more excess of UV light, which appears to be most dangerous to the retina and is detected by ventral S cones. Therefore ventral RPE can increase the local photoreceptor tolerance to a stress induced by excess of UV light when the mice are in the wild.

In summary, our results provide new evidence that the dorsal-central RPE is differentially prone to damage, and RPE degeneration in the dorsal visual field could be controlled by overlying M cones in mice. Hence, these findings have implications for a better understanding of the role of M cones in RPE regional degeneration-related diseases.

## Supplementary Material

Supplement 1

Supplement 2

Supplement 3

Supplement 4

Supplement 5

Supplement 6

## References

[1] Strauss O. The retinal pigment epithelium in visual function. *Physiol Rev**.* 2005; 85: 845–881. 15987797 10.1152/physrev.00021.2004

[2] Ma X, Li H, Chen Y, et al. The transcription factor MITF in RPE function and dysfunction. *Prog Retin Eye Res**.* 2019; 73: 100766. 31242455 10.1016/j.preteyeres.2019.06.002

[3] Cepko C. Intrinsically different retinal progenitor cells produce specific types of progeny. *Nat Rev Neurosci**.* 2014; 15: 615–627. 25096185 10.1038/nrn3767

[4] Kling A, Field G, Brainard D, Chichilnisky E. Probing Computation in the Primate Visual System at Single-Cone Resolution. *Annu Rev Neurosci**.* 2019; 42: 169–186. 30857477 10.1146/annurev-neuro-070918-050233 PMC6996509

[5] Lakkaraju A, Umapathy A, Tan L, et al. The cell biology of the retinal pigment epithelium [published online ahead of print February 24, 2020]. *Prog Retin Eye Res.*, doi:10.1016/j.preteyeres.2020.100846. PMC8941496 32105772

[6] Ambati J, Fowler B. Mechanisms of age-related macular degeneration. *Neuron*. 2012; 75: 26–39. 22794258 10.1016/j.neuron.2012.06.018 PMC3404137

[7] Fleckenstein M, Keenan T, Guymer R, et al. Age-related macular degeneration. *Nat Rev Dis primers**.* 2021; 7: 31. 33958600 10.1038/s41572-021-00265-2 PMC12878645

[8] Masri R, Grünert U, Martin P. Analysis of parvocellular and magnocellular visual pathways in human retina. *J Neurosci**.* 2020; 40: 8132–8148. 33009001 10.1523/JNEUROSCI.1671-20.2020 PMC7574660

[9] Peng Y, Shekhar K, Yan W, et al. Molecular classification and comparative taxonomics of foveal and peripheral cells in primate retina. *Cell**.* 2019; 176: 1222–1237.e1222. 30712875 10.1016/j.cell.2019.01.004 PMC6424338

[10] Weiter J, Delori F, Wing G, Fitch K. Retinal pigment epithelial lipofuscin and melanin and choroidal melanin in human eyes. *Invest Ophthalmol Vis Sci**.* 1986; 27: 145–152. 3943941

[11] Feeney-Burns L, Hilderbrand E, Eldridge S. Aging human RPE: morphometric analysis of macular, equatorial, and peripheral cells. *Invest Ophthalmol Vis Sci**.* 1984; 25: 195–200. 6698741

[12] Bermond K, Wobbe C, Tarau I, et al. Autofluorescent granules of the human retinal pigment epithelium: phenotypes, intracellular distribution, and age-related topography. *Invest Ophthalmol Vis Sci**.* 2020; 61: 35. 10.1167/iovs.61.5.35 PMC7405767 32433758

[13] Voigt A, Mulfaul K, Mullin N, et al. Single-cell transcriptomics of the human retinal pigment epithelium and choroid in health and macular degeneration. *P Natl Acad Sci USA**.* 2019; 116: 24100–24107. 10.1073/pnas.1914143116 PMC6883845 31712411

[14] Han S, Chen J, Hua J, et al. MITF protects against oxidative damage-induced retinal degeneration by regulating the NRF2 pathway in the retinal pigment epithelium. *Redox Biol**.* 2020; 34: 101537. 32361183 10.1016/j.redox.2020.101537 PMC7191850

[15] Kerur N, Fukuda S, Banerjee D, et al. cGAS drives noncanonical-inflammasome activation in age-related macular degeneration. *Nat Med**.* 2018; 24: 50–61. 29176737 10.1038/nm.4450 PMC5760363

[16] Kim J, Zhao H, Martinez J, et al. Noncanonical autophagy promotes the visual cycle. *Cell*. 2013; 154: 365–376. 23870125 10.1016/j.cell.2013.06.012 PMC3744125

[17] Kaarniranta K, Uusitalo H, Blasiak J, et al. Mechanisms of mitochondrial dysfunction and their impact on age-related macular degeneration. *Prog Retin Eye Res**.* 2020; 79: 100858. 32298788 10.1016/j.preteyeres.2020.100858 PMC7650008

[18] Bleckert A, Schwartz G, Turner M, Rieke F, Wong R. Visual space is represented by nonmatching topographies of distinct mouse retinal ganglion cell types. *Curr Biol**.* 2014; 24: 310–315. 24440397 10.1016/j.cub.2013.12.020 PMC3990865

[19] Baden T, Euler T, Berens P. Understanding the retinal basis of vision across species. *Nat Rev Neurosci**.* 2020; 21: 5–20. 31780820 10.1038/s41583-019-0242-1

[20] Warwick R, Kaushansky N, Sarid N, Golan A, Rivlin-Etzion M. Inhomogeneous encoding of the visual field in the mouse retina. *Curr Biol**.* 2018; 28: 655–665.e653. 29456141 10.1016/j.cub.2018.01.016 PMC6037286

[21] Gouras P, Ekesten B. Why do mice have ultra-violet vision? *Exp Eye Res**.* 2004; 79: 887–892. 15642326 10.1016/j.exer.2004.06.031

[22] Baden T, Schubert T, Chang L, et al. A tale of two retinal domains: near-optimal sampling of achromatic contrasts in natural scenes through asymmetric photoreceptor distribution. *Neuron*. 2013; 80: 1206–1217. 24314730 10.1016/j.neuron.2013.09.030

[23] Nadal-Nicolás F, Kunze V, Ball J, et al. True S-cones are concentrated in the ventral mouse retina and wired for color detection in the upper visual field. *eLife*. 2020; 9: e56840. 32463363 10.7554/eLife.56840 PMC7308094

[24] Kim Y, Yu H, Summers V, et al. Morphometric analysis of retinal pigment epithelial cells from C57BL/6J mice during aging. *Invest Ophthalmol Vis Sci**.* 2021; 62: 32. 10.1167/iovs.62.2.32 PMC7910641 33616620

[25] von Leithner P, Ciurtin C, Jeffery G. Microscopic mammalian retinal pigment epithelium lesions induce widespread proliferation with differences in magnitude between center and periphery. *Mol Vis**.* 2010; 16: 570–581. 20360994 PMC2847682

[26] Kokkinopoulos I, Shahabi G, Colman A, Jeffery G. Mature peripheral RPE cells have an intrinsic capacity to proliferate; a potential regulatory mechanism for age-related cell loss. *PloS One*. 2011; 6: e18921. 21526120 10.1371/journal.pone.0018921 PMC3081302

[27] Machalińska A, Lubiński W, Kłos P, et al. Sodium iodate selectively injuries the posterior pole of the retina in a dose-dependent manner: morphological and electrophysiological study. *Neurochem Res**.* 2010; 35: 1819–1827. 20725778 10.1007/s11064-010-0248-6 PMC2957578

[28] George S, Lu F, Rao M, Leach L, Gross J. The retinal pigment epithelium: Development, injury responses, and regenerative potential in mammalian and non-mammalian systems. *Prog Retin Eye Res**.* 2021; 85: 100969. 33901682 10.1016/j.preteyeres.2021.100969 PMC8536801

[29] Li H, Liu B, Lian L, et al. High dose expression of heme oxigenase-1 induces retinal degeneration through ER stress-related DDIT3. *Mol Neurodegener**.* 2021; 16: 16. 33691741 10.1186/s13024-021-00437-4 PMC7944639

[30] Fernandez-Godino R, Garland D, Pierce E. Isolation, culture and characterization of primary mouse RPE cells. *Nat Protoc**.* 2016; 11: 1206–1218. 27281648 10.1038/nprot.2016.065 PMC6432639

[31] Zheng Q, Tan Q, Ren Y, et al. Hyperosmotic stress-induced TRPM2 channel activation stimulates NLRP3 inflammasome activity in primary human corneal epithelial cells. *Invest Ophth Vis Sci**.* 2018; 59: 3259–3268. 10.1167/iovs.18-23965 29971445

[32] Li H, Lian L, Liu B, et al. KIT ligand protects against both light-induced and genetic photoreceptor degeneration. *eLife*. 2020; 9: e51698. 32242818 10.7554/eLife.51698 PMC7170656

[33] Li H, Fan L, Zhu S, et al. Epilation induces hair and skin pigmentation through an EDN3/EDNRB-dependent regenerative response of melanocyte stem cells. *Sci Rep-UK**.* 2017; 7: 7272. 10.1038/s41598-017-07683-x PMC5544680 28779103

[34] Zhao J, Kim H, Sparrow J. Multimodal fundus imaging of sodium iodate-treated mice informs RPE susceptibility and origins of increased fundus autofluorescence. *Invest Ophthalmol Vis Sci**.* 2017; 58: 2152–2159. 28395299 10.1167/iovs.17-21557 PMC5389744

[35] Moriguchi M, Nakamura S, Inoue Y, et al. Irreversible photoreceptors and RPE cells damage by intravenous sodium iodate in mice is related to macrophage accumulation. *Invest Ophthalmol Vis Sci**.* 2018; 59: 3476–3487. 30025075 10.1167/iovs.17-23532

[36] Hou L, Pavan W. Transcriptional and signaling regulation in neural crest stem cell-derived melanocyte development: do all roads lead to Mitf? *Cell Res**.* 2008; 18: 1163–1176. 19002157 10.1038/cr.2008.303

[37] Rózanowska M, Korytowski W, Rózanowski B, et al. Photoreactivity of aged human RPE melanosomes: a comparison with lipofuscin. *Invest Ophthalmol Vis Sci**.* 2002; 43: 2088–2096. 12091401

[38] Iwai-Takekoshi L, Ramos A, Schaler A, Weinreb S, Blazeski R, Mason C. Retinal pigment epithelial integrity is compromised in the developing albino mouse retina. *J Comp Neurol**.* 2016; 524: 3696–3716. 27097562 10.1002/cne.24025 PMC5063670

[39] Wang J, Iacovelli J, Spencer C, Saint-Geniez M. Direct effect of sodium iodate on neurosensory retina. *Invest Ophthalmol Vis Sci**.* 2014; 55: 1941–1953. 24481259 10.1167/iovs.13-13075 PMC4049579

[40] Chang L, Breuninger T, Euler T. Chromatic coding from cone-type unselective circuits in the mouse retina. *Neuron**.* 2013; 77: 559–571. 23395380 10.1016/j.neuron.2012.12.012

[41] Röhlich P, van Veen T, Szél A. Two different visual pigments in one retinal cone cell. *Neuron**.* 1994; 13: 1159–1166. 7946352 10.1016/0896-6273(94)90053-1

[42] Jiang Y, Qi X, Chrenek M, et al. Functional principal component analysis reveals discriminating categories of retinal pigment epithelial morphology in mice. *Invest Ophthalmol Vis Sci**.* 2013; 54: 7274–7283. 24114543 10.1167/iovs.13-12450 PMC4086880

[43] Xue Y, Shen S, Jui J, et al. CRALBP supports the mammalian retinal visual cycle and cone vision. *J Clin Invest**.* 2015; 125: 727–738. 25607845 10.1172/JCI79651 PMC4319437

[44] Xu H, Enemchukwu N, Zhong X, Zhang O, Fu Y. Deletion of M-opsin prevents M cone degeneration in a mouse model of Leber congenital amaurosis. *Am J Pathol**.* 2020; 190: 1059–1067. 32084365 10.1016/j.ajpath.2020.01.005 PMC7237827

[45] Böhm S, Splith V, Riedmayr L, et al. A gene therapy for inherited blindness using dCas9-VPR-mediated transcriptional activation. *Sci Adv**.* 2020; 6: eaba5614. 32875106 10.1126/sciadv.aba5614 PMC7438099

[46] Chowers G, Cohen M, Marks-Ohana D, et al. Course of sodium iodate-induced retinal degeneration in albino and pigmented mice. *Invest Ophthalmol Vis Sci**.* 2017; 58: 2239–2249. 28418497 10.1167/iovs.16-21255

[47] Gibbs D, Azarian S, Lillo C, et al. Role of myosin VIIa and Rab27a in the motility and localization of RPE melanosomes. *J Cell Sci**.* 2004; 117: 6473–6483. 15572405 10.1242/jcs.01580 PMC2942070

[48] Rózanowski B, Burke J, Boulton M, Sarna T, Rózanowska M. Human RPE melanosomes protect from photosensitized and iron-mediated oxidation but become pro-oxidant in the presence of iron upon photodegradation. *Invest Ophthalmol Vis Sci**.* 2008; 49: 2838–2847. 18326697 10.1167/iovs.08-1700

[49] Olchawa M, Szewczyk G, Zadlo A, Sarna M, Wnuk D, Sarna T. The effect of antioxidants on photoreactivity and phototoxic potential of RPE melanolipofuscin granules from human donors of different age. *Antioxidants**.* 2020; 9: 1044. 10.3390/antiox9111044 PMC7693403 33114498

[50] Enzbrenner A, Zulliger R, Biber J, et al. Sodium iodate-induced degeneration results in local complement changes and inflammatory processes in murine retina. *Int J Mol Sci**.* 2021; 22: 9218. 34502128 10.3390/ijms22179218 PMC8431125

