# Supplementary material for: Sensitivity of the Dorsal-Central Retinal Pigment Epithelium to Sodium Iodate-Induced Damage Is Associated With Overlying M-Cone Photoreceptors in Mice
Source: Invest Ophthalmol Vis Sci. 2022 Aug 26;63(9):29. doi: 10.1167/iovs.63.9.29 (PMC9428360; doi:10.1167/iovs.63.9.29)
Supplement: Supplement 1 [file iovs-63-9-29_s001.pdf]

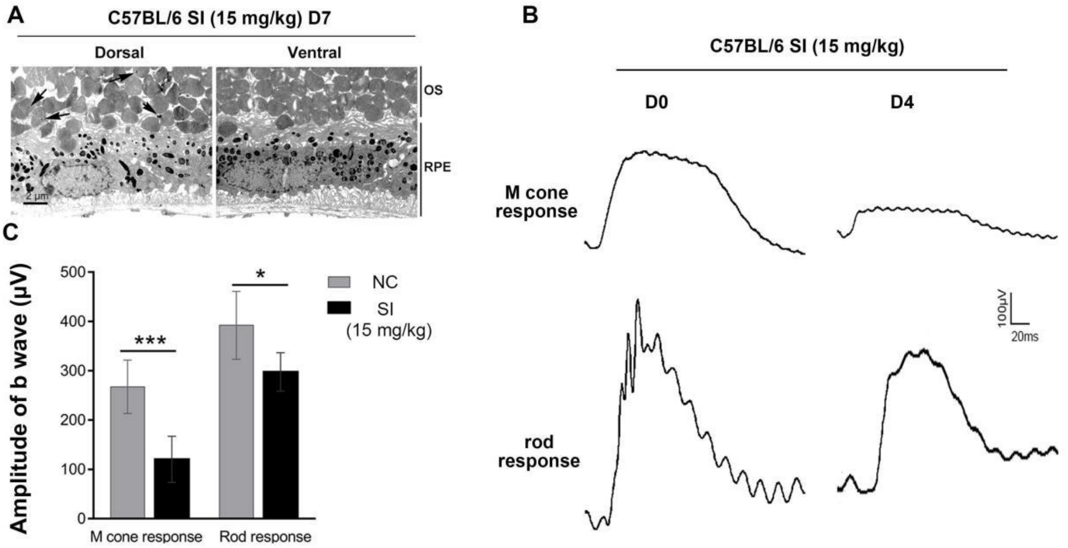

Fig S1. Low dose of SI induces mislocalization of melanin granules in C57BL/6 dorsal retinas at day 7 and reduces retinal function of C57BL/6 mice at day 4. **(A)** Distribution of melanosomes in the dorsal and ventral retinas after 7 days of single low dose SI (15 mg/kg) injection was revealed by transmission electron microscopy (TEM). The black arrows point to the mislocalization of melanin granules in the OS. n=6; Scale bars, 2 μm. **(B)** ERG traces of the M-cone (Upper panels) and rod responses (lower panels) from C57BL/6 mice injected with a low dose SI at day 0 or day 4 were elicited by green light with a strength of 0.75 cd-s/m^2 and scotopic light with strength of 0.01cd-s/m^2, respectively. **(C)** The bar graphs show the quantification of the amplitude of b-wave from the M-cone response and rod response. n=5.*P<0.05; ***P<0.001. Data are presented as the mean ± standard error of the mean and were compared using a student's t-test. OS, photoreceptor outer segment; NC, Normal control; SI, sodium iodate.
