# Supplementary material for: Sensitivity of the Dorsal-Central Retinal Pigment Epithelium to Sodium Iodate-Induced Damage Is Associated With Overlying M-Cone Photoreceptors in Mice
Source: Invest Ophthalmol Vis Sci. 2022 Aug 26;63(9):29. doi: 10.1167/iovs.63.9.29 (PMC9428360; doi:10.1167/iovs.63.9.29)
Supplement: Supplement 2 [file iovs-63-9-29_s002.pdf]

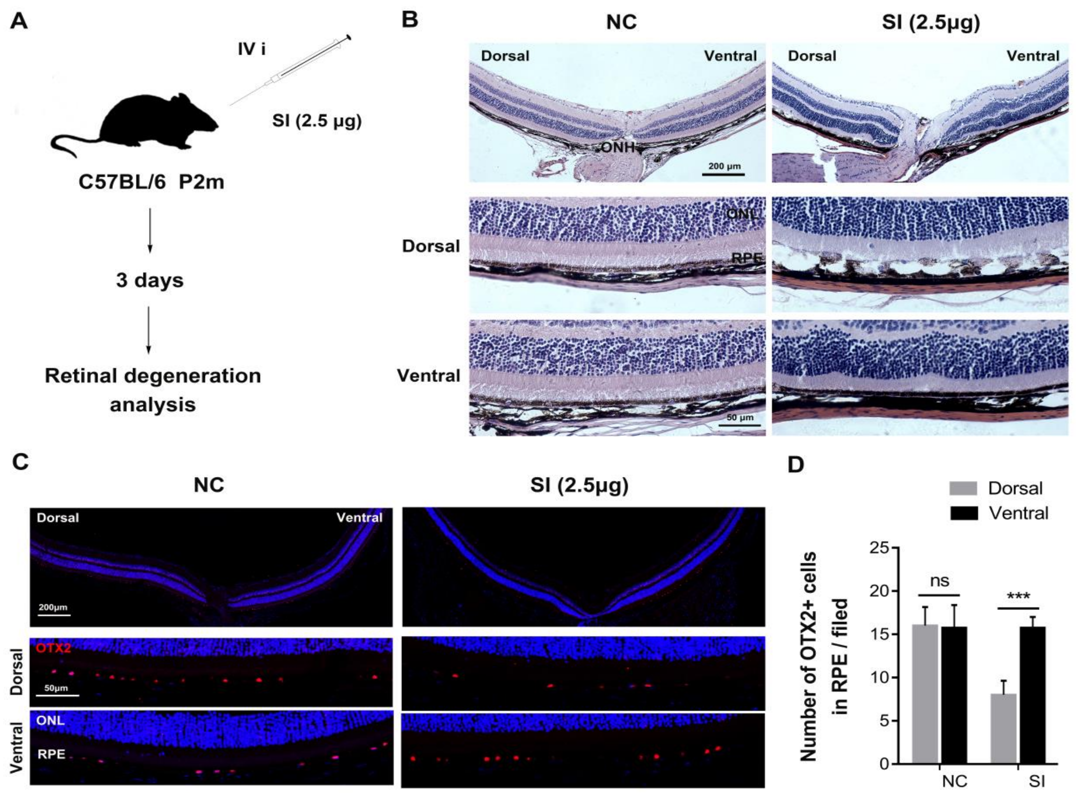

Fig S2. Induced degeneration in dorsal RPE by intravitreal injection of SI. **(A)** Schematic representation of the experimental procedure of SI injury. A 2-month-old C57BL/6 mouse was injected with a dose SI (2.5 μg) via the intravitreal injection, and retinal degeneration analysis was performed on day 3 after the single injection. **(B)** The histological structure of retinas from mice after 3 days of SI or saline injection. Scale bar, 200 μm (upper panel) and 50 μm (middle panel). **(C)** Immunodetection of OTX2 in the RPE of C57BL/6 mice under the indicated conditions. **(D)** Quantification of the OTX2 positive cells in the dorsal and ventral RPE from the C57BL/6 mice injected with SI or saline. n=6. ***P<0.001. Data are presented as the mean ± standard error of the mean and were compared using a student's t-test. ONL, outer nuclear layer; RPE, retinal pigment epithelium.
