# Supplementary material for: Sensitivity of the Dorsal-Central Retinal Pigment Epithelium to Sodium Iodate-Induced Damage Is Associated With Overlying M-Cone Photoreceptors in Mice
Source: Invest Ophthalmol Vis Sci. 2022 Aug 26;63(9):29. doi: 10.1167/iovs.63.9.29 (PMC9428360; doi:10.1167/iovs.63.9.29)
Supplement: Supplement 3 [file iovs-63-9-29_s003.pdf]

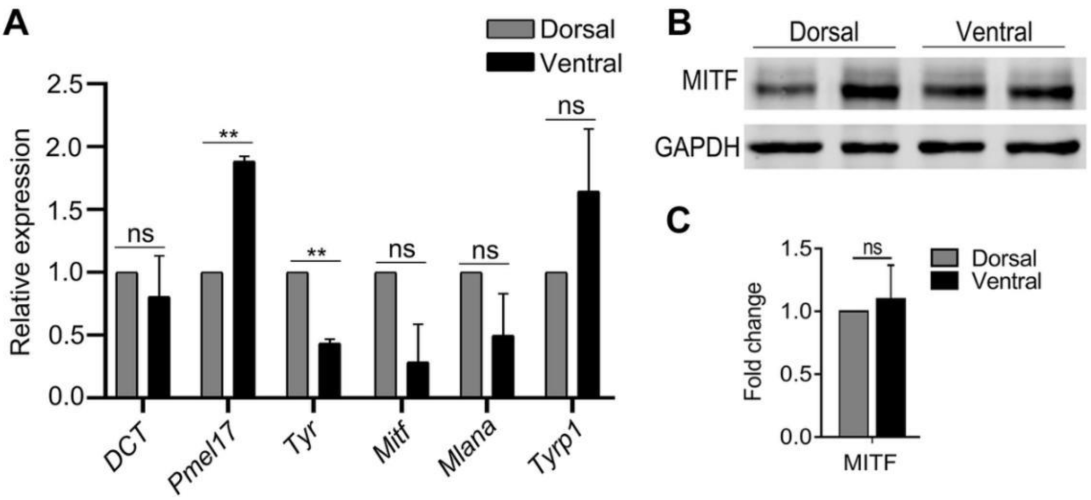

Fig S3. Expression of melanin synthesis-related genes in dorsal and ventral RPE. (**A**) Q-PCR was performed to analyze transcript differences of melanin synthesis-related genes between dorsal and ventral RPE from the 2-month-old C57BL/6 mice. n=5. $^{**}P < 0.01$ and $^{*}P < 0.05$. Data are presented as the mean ± standard error of the mean and were compared using a student's t-test. (**B**) Western blots showed MITF expression in the dorsal and ventral RPE from the adult C57BL/6 mice. (**C**) Quantification of MITF protein level in dorsal and ventral RPE based on the western blot analysis. n=4. ns, not significant. Data are presented as the mean ± standard error of the mean and were compared using a student's t-test.
