# Supplementary material for: Sensitivity of the Dorsal-Central Retinal Pigment Epithelium to Sodium Iodate-Induced Damage Is Associated With Overlying M-Cone Photoreceptors in Mice
Source: Invest Ophthalmol Vis Sci. 2022 Aug 26;63(9):29. doi: 10.1167/iovs.63.9.29 (PMC9428360; doi:10.1167/iovs.63.9.29)
Supplement: Supplement 5 [file iovs-63-9-29_s005.pdf]

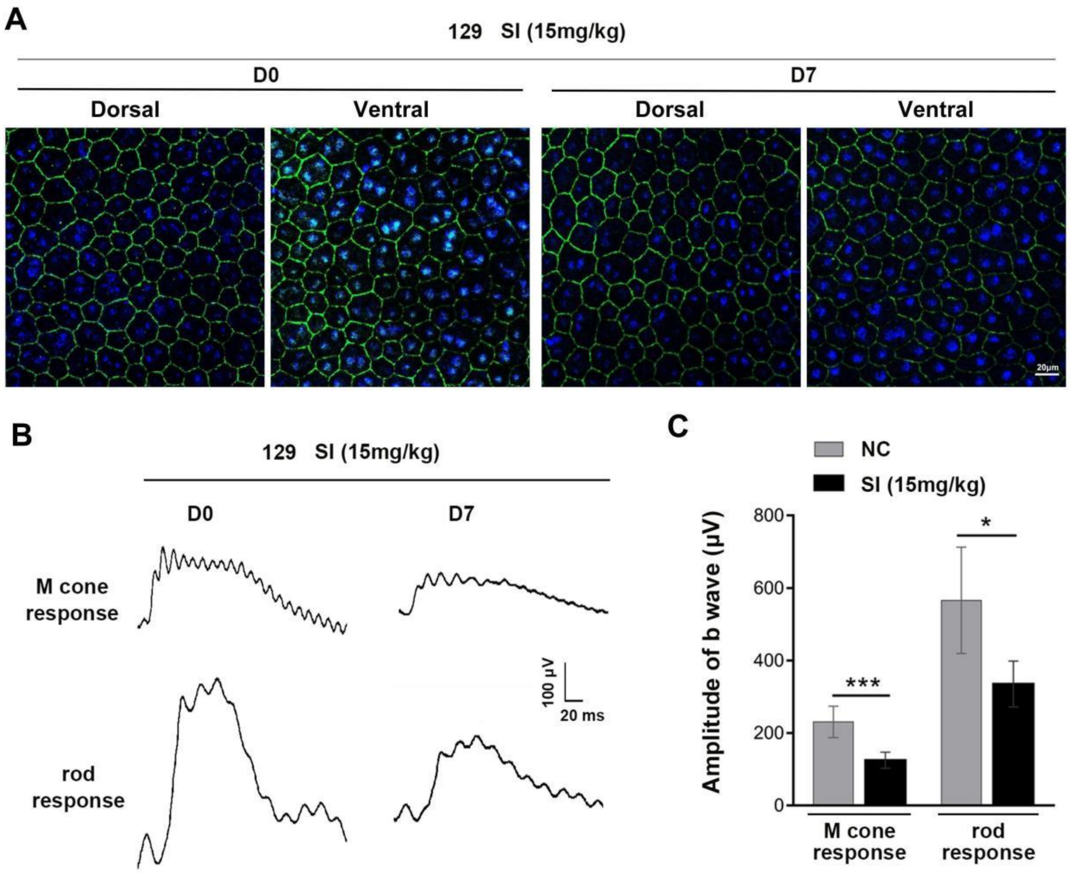


**Fig S5.** The low dose of SI does not induce RPE degeneration but significantly reduces retinal function in albino mice. **(A)** Immunostaining images of anti-ZO1 in the flat-mounted RPE of the 2-month-old 129 mice 7 day after a single injection of saline or low dose SI (15 mg/kg). Scar bar, 20 μm. **(B)** ERG traces of the M-cone (upper panels) and rod responses (lower panels) from 129 mice injected with a low dose SI at day 0 or day 7 were elicited by green light with a strength of 0.75 cd-s/m$^2$ and scotopic light with a strength of 0.01cd-s/m$^2$, respectively. **(C)** The bar graphs show the quantification of the amplitude of the b-wave from the M-cone response and rod response. n=5. *P<0.05; ***P<0.001. Data are presented as the mean ± standard error of the mean and were compared using a student's t-test. SI, sodium iodate.
