# Supplementary material for: Sensitivity of the Dorsal-Central Retinal Pigment Epithelium to Sodium Iodate-Induced Damage Is Associated With Overlying M-Cone Photoreceptors in Mice
Source: Invest Ophthalmol Vis Sci. 2022 Aug 26;63(9):29. doi: 10.1167/iovs.63.9.29 (PMC9428360; doi:10.1167/iovs.63.9.29)
Supplement: Supplement 6 [file iovs-63-9-29_s006.pdf]

Supplemental table

Table S1. The primer sequences for detecting expression of melanin-synthesis related genes.

Gene (ID)	Forward	Reverse
Mitf (17342)	CCTTCCAGCGTTTCTATGTC	ACTTCTTTCAGTTTGGGTTA
Dct(13190)	GTCCTCCACTCTTTTACAGACG	ATTCGGTTGTGACCAATGGGT
Tyr(22173)	CTCTGGGCTTAGCAGTAGGC	GCAAGCTGTGGTAGTCGTCT
Tyrp1(22178)	ATGAAATCTTACAACGTCCTCCC	GCACACTCTCGTGGAAACTGA
Pmel17(20431)	TGGCAGAGGTGTCAACTA	CATTATGGTGTCGGTGTCA
Mlana(77836)	CTGTAGAAGACGAAGTGGAT	GAATAAGGTGGCGGTGAA
Gapdh(14433)	AGGTCGGTGTGAACGGATTTG	TGTAGACCATGTAGTTGAGGTCA
